# Insights of Phage-Host Interaction in Hypersaline Ecosystem through Metagenomics Analyses

**DOI:** 10.3389/fmicb.2017.00352

**Published:** 2017-03-03

**Authors:** Amir Mohaghegh Motlagh, Ananda S. Bhattacharjee, Felipe H. Coutinho, Bas E. Dutilh, Sherwood R. Casjens, Ramesh K. Goel

**Affiliations:** ^1^Department of Civil and Environmental Engineering, University of UtahSalt Lake, UT, USA; ^2^Instituto de Biologia, Universidade Federal do Rio de JaneiroRio de Janeiro, Brazil; ^3^Radboud Institute for Molecular Life Sciences, Centre for Molecular and Biomolecular Informatics, Radboud University Medical CentreNijmegen, Netherlands; ^4^Theoretical Biology and Bioinformatics, Utrecht UniversityUtrecht, Netherlands; ^5^Department of Pathology, University of UtahSalt Lake, UT, USA

**Keywords:** bacteriophage, phageome, viromics, phage-host interaction, prophage, phage-host network

## Abstract

Bacteriophages, as the most abundant biological entities on Earth, place significant predation pressure on their hosts. This pressure plays a critical role in the evolution, diversity, and abundance of bacteria. In addition, phages modulate the genetic diversity of prokaryotic communities through the transfer of auxiliary metabolic genes. Various studies have been conducted in diverse ecosystems to understand phage-host interactions and their effects on prokaryote metabolism and community composition. However, hypersaline environments remain among the least studied ecosystems and the interaction between the phages and prokaryotes in these habitats is poorly understood. This study begins to fill this knowledge gap by analyzing bacteriophage-host interactions in the Great Salt Lake, the largest prehistoric hypersaline lake in the Western Hemisphere. Our metagenomics analyses allowed us to comprehensively identify the bacterial and phage communities with *Proteobacteria, Firmicutes*, and *Bacteroidetes* as the most dominant bacterial species and *Siphoviridae, Myoviridae*, and *Podoviridae* as the most dominant viral families found in the metagenomic sequences. We also characterized interactions between the phage and prokaryotic communities of Great Salt Lake and determined how these interactions possibly influence the community diversity, structure, and biogeochemical cycles. In addition, presence of prophages and their interaction with the prokaryotic host was studied and showed the possibility of prophage induction and subsequent infection of prokaryotic community present in the Great Salt Lake environment under different environmental stress factors. We found that carbon cycle was the most susceptible nutrient cycling pathways to prophage induction in the presence of environmental stresses. This study gives an enhanced snapshot of phage and prokaryote abundance and diversity as well as their interactions in a hypersaline complex ecosystem, which can pave the way for further research studies.

## Introduction

Microbial communities and particularly prokaryotes provide diverse ecosystem functions in aquatic habitats. These functions include the cycling of carbon (Jasser et al., 2009; Li et al., 2010), sulfur (Sorokin et al., 2012), nutrients (Kouki et al., 2009; Wang et al., 2013; Motlagh and Goel, 2014), mercury transformation (Chavan et al., 2007; Mitchell et al., 2009; Ramasamy et al., 2012), sulfate reduction (Fortin et al., 2000; Bahr et al., 2005; Faulwetter et al., 2013), and many other biological processes. Therefore, the study of aquatic microbial communities can provide important information about nutrient removal processes, ecosystem functioning and drivers of biodiversity (Gough and Stahl, 2010; Kim et al., 2011).

Bacteriophages outnumber prokaryotic cells in many ecosystems, thus exerting significant predation pressure on their hosts (Williamson et al., 2008). This pressure plays a critical role in the evolution, diversity, and abundance of prokaryotes (Stern and Sorek, 2011). Bacteriophages infect and lyse up to 40% of the prokaryotic population in marine sediments on a daily basis (Middelboe, 2008), resulting in decay of the cell mass and affecting the carbon pool in sediments, which causes more nutrients to be released into the water column. In addition, bacteriophages can influence the genetic diversity of prokaryotic communities in many different ways. Phages selectively kill their hosts, often in a “kill-the-winner” dynamic (Thingstad et al., 2008) in which the most abundant members of a microbial community are the most targeted by phage infection, consequently having their genes temporarily depleted from the genetic pool of a given habitat (Motlagh et al., 2016).

Phages also contribute to the genetic diversification of prokaryotic communities through transduction, a form of phage-mediated horizontal gene transfer (HGT). Through HGT organisms can acquire exogenous DNA from closely or distantly related lineages, and recognition of this process has caused a shift from a diverging “tree of life” view to a “web of life” view of evolution (Olendzenski and Gogarten, 2009; Puigbó et al., 2010). Between 1.6 and 32.6% of genes in prokaryote genomes are estimated to have been acquired through horizontal gene transfer (Koonin et al., 2001), while up to 81% of the genes are estimated to be affected in prokaryotic genomes if the cumulative impact of horizontal gene transfer toward a lineage is also considered (Dagan et al., 2008). Transduction has been studied in various natural environments including freshwater (Kenzaka et al., 2010), marine (Jiang and Paul, 1998), plant-associated (Kidambi et al., 1994), and wastewater systems (Del Casale et al., 2011). However, most previous work has been focused on understanding phage and prokaryote genomes in the transduction process, while the role of transduction in the diversification of prokaryotic genomes and how it affects ecological processes driven by prokaryotes remains poorly understood. This is of special relevance considering the previous research studying the susceptibility of marine microbes to infection by phages isolated from soil, marine sediments, and fresh water and demonstrating that phages move and propagate between major biomes, mediating the transfer of DNA between microbes from very different ecosystems (Sano et al., 2004).

An important component in the study of phage-host interactions is the symbiotic state known as lysogeny, i.e., the stable and non-lytic co-existence of a whole viral genome inside the prokaryotic host genome. Such quiescent phage genomes are called prophages, and they can be integrated into the chromosome of the host bacterium or exist as plasmids. Prophages often account for most of the difference between strains of the same microbial species (Casjens, 2003; Dutilh et al., 2014), and in a “piggyback-the-winner” strategy prophages may become especially important in high microbial density situations (Knowles et al., 2016). Although the integration of temperate phages into the host genome can be beneficial to host and phage (Yosef et al., 2015), these prophages may be induced by a wide range of environmental stress factors such as nutrients (McDaniel and Paul, 2005) or heavy metals (Motlagh et al., 2015) which leads to lysis of the bacterial cell and phage virion release. Moreover, a prophage can dramatically change the phenotype of the host via lysogenic conversion (Paul, 2008). Therefore, studying the presence and role of prophages in natural ecosystems is a critical step toward understanding phage-host interactions.

Studies over the previous decades have greatly increased estimations of the genetic richness of viruses in aquatic environments. Culture-independent techniques such as restriction fragment length polymorphism (RFLP) (Chen et al., 1996) and PCR-based methods such as denaturing-gradient-gel electrophoresis (DGGE) (Short and Suttle, 1999), pulse-field-gel electrophoresis (PFGE) (Bhattacharjee et al., 2015), and hybridization analyses (Wichels et al., 1998; Wommack et al., 1999) provided initial snapshots of diversity among prokaryotic communities. However, understanding the taxonomic viral diversity in the environment is challenging since, unlike bacteria, viruses do not carry universally conserved genetic elements (e.g., ribosomal RNA genes of bacteria) that could be used as taxonomic markers to identify all viruses (Rohwer and Edwards, 2002). Metagenomics sequencing approaches have enabled exploration of genetic diversity within environmental samples, thus evading limitations associated with conventional culture-dependent microbiology methods. In addition, by targeting total nucleic acids, shotgun metagenomics allows functional and taxonomic characterization of samples without the need for prior knowledge of the microbial types present in the studied environment.

Previous studies have demonstrated the effect of phages and their infection processes on the sediment microbiome in different environments such as wetland (Jackson and Jackson, 2008), marine (Rohwer and Thurber, 2009), and freshwater (Short and Suttle, 2005). However, hypersaline environments are among the least studied ecosystems, and so there is a lack of knowledge about the interaction between prokaryotic communities and bacteriophages in such habitats. The Great Salt Lake (GSL) is the largest prehistoric hypersaline lake in the Western Hemisphere, having a unique geology that gives rise to special ecologic and economic domains of relevance in its various uses as both a migratory bird habitat and a source of brine shrimp, trace elements, and other minerals. In the present study, we employed metagenomics analysis to determine the diversity of phages and prokaryotes in the GSL, as well as to better understand the effects of bacteriophages in defining the diversity and interactions among prokaryotic communities and their biogeochemical cycles.

## Materials and methods

### Site description and sediment sampling

The Great Salt Lake (GSL) is a terminal lake that represents a complex hypersaline ecosystem with a salinity gradient between 6 and 28% that is contaminated with, for example, mercury, selenium, and nutrients (Williams, 2002). The lake is fed by the Weber, Bear, and Jordan rivers. These rivers carry more than 1.1 million tons of salts annually into the lake (Rafferty, 2011), which transformed GSL into one of the most saline bodies of water in the world. The total dissolved mineral accumulation in the lake basin is estimated at 5 billion tons, consisting of mainly sodium, and chloride ions, though sulfate, magnesium, and potassium are also abundant (Stephens, 1998). The sediment sample was collected 6 miles west of Antelope Island (40°53'51.3” N, 112°21'00” W) in the South Arm of the Great Salt Lake (Figure S1). Physicochemical data in the water column were obtained using a sounder equipped with pH, specific conductivity, water temperature, depth, and dissolved oxygen sensors. The sampling was carried out in June 2014 with the collaboration of the United States Geological Survey (USGS). Sediment sample was collected at a depth of 27 ft from the deep brine layer at the surface of the sediment with a stainless steel box corer (Wildco, FL). The sediment sample was immediately stored on ice and shipped to the laboratory for analysis.

### Characteristics of the environmental sample

For nutrient determination, sediment sample was centrifuged at 2,000 × g for 10 min to extract interstitial and pore water. The pore water was filtered with 0.45 μm mixed cellulose hydrophilic filter paper (Millipore, MA) and ammonia (NH_3_), nitrite (NO_2_), nitrate (${\text{NO}}_{3}^{-}$), total nitrogen (TN), total phosphorus (TP), and total organic carbon (TOC) were quantified using HACH methods HACH 8155, TNT839, TNT835, TNT826, TNT843, and HACH 10128, respectively, according to the manufacturer's instructions. In order to ensure that the high salinity of the samples did not interfere with the measurement procedure and reading the spectrophotometer, control samples with known concentration of ammonia, nitrite, nitrate, and phosphorus were also used alongside each sample. Total solids (TS) and volatile solids (VS) were also measured by drying the sediment sample at 103°C for 24 h followed by igniting at 550°C for 2 h according to EPA Method 1684 (USEPA, 2001). In addition, to study the biogeochemistry of the sediment sample, trace metal concentrations (Mn, Fe, Co, Ni, Cu, Zn, Se, Mo, and Pb) were measured by using 15 g of sediment, centrifuging at 5,000 × g for 10 min and filtering the supernatant with 0.22 μm hydrophilic filter and then analyzed using inductively coupled plasma mass spectrometry (ICP-MS, Agilent 7500ce, CA).

### Microbial nucleic acid extraction

Five grams of sediment were re-suspended in sterilized 10 ml of PBS buffer and vortexed for 5 min to homogenize the sediment. The homogenized sample was then centrifuged for 5 min at 2,000 × g and 250 μl of clear supernatant devoid of any cells was discarded. The pellet was re-suspended in 1 mL of PBS buffer and 500 μl of sample was used for DNA extraction with different methods including phenol-chloroform DNA extraction (Köchl et al., 2005), cetyl trimethyl ammonium bromide (CTAB) extraction (Zhou et al., 1996), PowerSoil® DNA isolation kit (MoBio laboratories, CA), and PowerMax™ Soil DNA isolation kit (MoBio laboratories, CA) following the manufacturers' procedures. In order to minimize the extraction biases, the extracted DNA was pooled and the quantity and quality were verified on a NanoDrop ND 2,000 spectrophotometer (Thermo Scientific, USA) at 260 and 280 nm. The DNA purity and quantity were also verified on 1.2% agarose gel prior to high-throughput sequencing.

### Bacteriophage isolation and nucleic acid extraction

Free phages were extracted by re-suspending 250 g of sediment in 3 × volume of filter sterilized 1% (w/v) potassium citrate buffer (10 g/l potassium citrate, 1.44 g/l of disodium phosphate, 0.24 g/l of monopotassium phosphate, pH 7). The sediment with potassium citrate buffer was placed on a shaker on ice overnight to suspend free phages in solution. Thereafter, the mixture was centrifuged at 8,000 rpm on an Avanti J-E centrifuge (Beckman Coulter, CA) for 30 min to pellet the bacterial debris followed by centrifugation of the supernatant at 9,000 rpm for 12 h to concentrate the phages. Following overnight centrifugation, the pellet was re-suspended in SMG buffer (5.8 g/l sodium chloride, 2 g/l magnesium sulfate, 5 ml/l of 5% (w/v) gelatin, 50 ml/l of 1M Tris–Cl, pH 7.5) and filtered with 0.22 μm pore size filter paper (Millipore Co., MA) to remove any residual bacterial cells and sediment debris.

The phage particles were purified using cesium chloride (CsCl) gradients at 1.35–1.6 g/ml density by isopycnic centrifugation at 35,000 rpm for 3 h (Angly et al., 2006). This process was carried out twice to ensure the removal of any residual bacterial cell debris and guarantee the purity of viral particles. Before DNA extraction from the virions, the sample was subjected to DNase treatment with RNase-free DNase I (Thermo Scientific, CA) at 37°C for 30 min. This step digested any free DNAs in the sample. Subsequent phage DNA extraction was performed with a phage DNA isolation kit (Norgen Biotek Corp., Canada). In addition, to confirm the absence of microbial DNA contamination in the phage DNA extracts, PCR amplification of the hypervariable V4–V9 region was tested using 515F/1492R universal 16S rRNA gene primer set (Diemer and Stedman, 2012). Aliquots of the amplification products were electrophoresed in a 1.2% agarose gel stained with ethidium bromide (10 μg/ml) and visualized under UV illumination.

### DNA library preparation for sequencing

Amplification methods such as random amplified shotgun library (RASL), linker-amplified shotgun library (LASL), and multiple displacement amplification (MDA) are usually implemented to increase the nucleic acid yield and obtain a sufficient quantity of DNA for sequencing. However, these methods can result to quantitative biases such as selective amplification of single-stranded DNA (ssDNA) viruses' genomes (Kim et al., 2008) and production of artifacts such as chimeras (Lasken and Stockwell, 2007). Therefore, we refrained from using any amplification process in order to minimize biases for the phage metagenomics and analysis of the phageome data.

Library construction was performed using the Epicentre EpiGnome Methyl-Seq Kit as described below. Briefly, genomic DNA (~50 ng) was heat denatured and hybridized with oligonucleotides consisting of random hexamers linked to Illumina P5 adapter sequences. Strand replication was accomplished using EpiGnome polymerase. Double-stranded DNA was heat denatured to enable ligation of the EpiGnome Terminal Tagging Oligo which adds Illumina P7 adapter sequence to the 3′ end of the replicated strand. Adapter-ligated DNA molecules were enriched by 10 cycles of PCR and the amplified library was subsequently purified using Agencourt AMPure XP beads (Beckman Coulter Genomics, CA). The concentration of the library was measured using the Qubit dsDNA HS Assay (Invitrogen, CA) and an aliquot of the library was resolved on an Agilent 2,200 Tape Station using a D1000 assay to define the size distribution of the sequencing library. Libraries were adjusted to a concentration of approximately 10 nM and quantitative PCR was performed using the Kapa Library Quant Kit (Kapa Biosystems, MA) to calculate the molarity of adapter-ligated DNA molecules. The concentration was further adjusted following qPCR to prepare the library for Illumina sequence analysis and the samples were sequenced on an Illumina MiSeq Bench top DNA sequencer (Illumina, CA) with 300-cycles paired-end at HCI Core Facility of University of Utah.

### Metagenomic analysis

Paired-end raw reads were interleaved, quality filtered and trimmed using CLC Genomics Workbench v.7.0.4 (CLC Bio, Denmark) with a threshold of 100 bp as the minimum length of read and a Phred score of 28. The trimmed reads were *de novo* assembled using CLC Genomics Workbench v.7.0.4 with the following criteria: word size of 20 bp, automatic bubble size of 50 bp, and minimum contig length of 500 bp. Identification of open reading frames (ORFs) and gene prediction were performed using MetaGeneMark v.2.8 (Zhu et al., 2010) followed by gene annotation using the RPSBLAST program (Altschul et al., 1990) on the clusters of orthologous group (COG) prokaryotic protein database (Tatusov et al., 2000). Statistical over-representation of annotated COG genes and Kyoto Encyclopedia of Genes and Genomes (KEGG) pathways was determined by pairwise comparisons of each metagenomic sample using Fisher's exact test, with confidence intervals at 99% significance. Besides COG analysis, the predicted genes were categorized into SEED Subsystems to provide consistent and accurate genome annotations (Overbeek et al., 2014) through MG-RAST (metagenomics rapid annotation using subsystem technology) pipeline to identify all of the encoded proteins and their functions in the host metabolism. The enzymes were compared against SEED Subsystems using a maximum *e*-value of 1E-5, a minimum identity of 60%, and a minimum alignment length of 100 measured in amino acids for protein databases. The minimum alignment length of 100 amino acids (equal to 300 bps) was chosen as the criterion to specify the possible functional genes (not only partial genes) that are transferred from prokaryotes to the phages.

In order to understand the phage-host interactions, the GC contents of phage and prokaryote contigs were calculated and compared with contig percentage shown in a histogram. The occurrence of each single tetranucleotide in the phage and prokaryote contigs was calculated using JSpecies v.1.2.1 (Richter and Rossello-Mora, 2009). In addition, the calculated GC content of phage and prokaryote contigs were graphed along with the scaffold length and contig coverage using the ggplot package in R v.3.0.1. The sequences are deposited in MG-RAST repository with accession number mgm4582011.3 and mgm4582012.3 for bacteria and phage sequences, respectively.

#### Analysis of prokaryotic metagenome

The taxonomic composition of the prokaryotic metagenome was determined by comparison of the assembled contigs against the NCBI non-redundant (nr) nucleotide database using tBLASTx v. 2.2.28 program (Altschul et al., 1990) with 1E-5 *e*-value cut-off. Relative abundance of taxa was assessed on the basis of average counts of mapped reads on the BLAST annotated contigs. Therefore, for the reads that were mapped to contigs, every blast hit was multiplied by the average depth of the reads on each contig. Eventually, significant hits to GenBank entries were recorded in a BLAST output file and imported on MEGAN v.5 (Huson et al., 2007) for interpretation with the lowest common ancestor (LCA) method followed by visualization in iTOL online tool (Letunic and Bork, 2007). For the MEGAN analysis, default parameters for each metagenome were selected as follows: minimum support of 5, minimum score of 50, top percent of 10, win-score of 0, and minimum complexity of 0.44.

The affinities of the sequences for known metabolic functions were also annotated using BLASTx with cut-off *e*-value of 1E-5 against SEED subsystems v.2.0 (Overbeek et al., 2005) using MG-RAST server v.3.3 (Meyer et al., 2008) and KEGG metabolic pathways (Kanehisa et al., 2008). In addition, to understand the role of prokaryotic communities in nutrient cycles, the identified prokaryotes were compared manually with a curated database on functional genes (FunGene) involved in various biogeochemical cycles (Fish et al., 2013).

#### Analysis of phage metagenome

In addition to initial viral particle purification step prior to sequencing and DNase treatment of the purified phage prior to DNA extraction, in order to exclude any bacterial contamination from the phage contigs, genes encoding the 5S, 16S, and 23S rRNAs (from prokaryotic genomes) were identified in phage contigs using hmm_rRNA on WebMGA server based on an HMM search using HMMER v.3 (Finn et al., 2011).

Since analysis of the phage metagenome was also performed on contigs instead of reads, the relative abundance of contigs was corrected by the average depth of the reads on each contig as mentioned earlier. For phage taxonomic analysis, the filtered phage contigs were compared against NCBI RefSeq viral genome database by using tBLASTx v. 2.2.28 program with *e*-value of 1E-5 cut-off. The generated BLAST output file was imported on MEGAN v.5 for interpretation by lowest common ancestor (LCA) method using the default parameters as mentioned above (results not shown). Instead, the abundance of phages from the GSL was explored with respect to metagenomes of marine, freshwater, and lake sediment samples. Raw reads of aquatic viromes available on Metavir (Roux et al., 2011) were mapped to assembled phage contigs with length longer than 20 kbp as putative complete phages using Bowtie v.2 (Langmead and Salzberg, 2012). Contig abundances were obtained by counting the number of reads mapped to each contig and correcting by contig length. The obtained abundance matrix with identity percentages was normalized and plotted as a heatmap in which samples and contigs were clustered according to their Euclidean distance using R v.3.0.1.

#### Prophage identification and analysis

To achieve a more detailed understanding of the phage-host interactions, mobile genetic elements including prophages, plasmids, and transposons were investigated in the prokaryote contigs. Following gene prediction in the prokaryote contigs, integrated prophages were detected using Prophinder (Lima-Mendez et al., 2008) to identify mobile genetic elements. Simultaneously, in order to relate the prophages to their host, prokaryote contigs were compared against ACLAME (A CLAssification of Mobile genetic Elements) database (Leplae et al., 2004) using a BLASTn search with *e*-value 1E-5 cut-off, followed by extraction of the prophage hit regions (excluding viruses and plasmids) from the BLAST output results. These prophage regions were compared with prophages found using Prophinder and the shared prophages with their corresponding prokaryotic hosts were extracted to generate the taxonomic cladogram using MEGAN with default parameters mentioned earlier. In addition, nutrient cycling of the prokaryotic hosts was manually annotated based on the KEGG database and used to generate the prophage-host interaction network using iTOL online tool.

#### Clustered regularly interspaced short palindromic repeat (CRISPR)

Clustered regularly interspaced short palindromic repeat (CRISPR) is an anti-viral mechanism in archaea and bacteria wherein genomic sequences from the predatory viruses are integrated in the host genome providing immunity to these viruses (Horvath and Barrangou, 2010). Therefore, CRISPR spacer sequences can provide a direct link between viruses and their prokaryotic hosts (Kunin et al., 2008; Heidelberg et al., 2009; Anderson et al., 2011) and CRISPR spacers may be viewed as a database of fragments derived from phage and plasmid genomes. In addition, phage genome can acquire some prokaryotic genes during an infection event of the host and therefore, homology of phage and prokaryotic genes can indicate phage-host associations. In our study, the assembled bacteria contigs were compared against phage contigs as the database by using tBLASTx v. 2.2.28 setting a 1E-5 *e*-value cut-off to find their homology. Afterward, all CRISPR arrays in the prokaryote contigs with homology to phage contigs were identified using CRISPR Recognition Tool (CRT) v.1.1 (Bland et al., 2007). Putative protospacer targets were also identified using CRISPRTarget (Biswas et al., 2013), following a BLASTn search of the spacer input against ACLAME, GenBank Environmental, GenBank Phage, and RefSeq plasmid, RefSeq viral databases with the default settings.

## Results

### Characteristics of the environmental sample

Following sample collection from 27-ft deep brine layer, the environmental quality parameters, nutrients, and metals of the sediment sample were measured as summarized in Table 1. Measurement of nutrient and metal concentrations in the sediment can be an indicator of microbial activities involved in different nutrient cycles. Salinity was measured as the total salt concentration, comprised mostly of Na^+^ and Cl^−^ ions. The measured salinity of the GSL was 15% (150 g/l), which is approximately 5 times the average salinity of the ocean and 300 times the average salinity of fresh water (Kerr et al., 2003). In addition, significantly low dissolved oxygen levels in the deep brine layer and high concentration of total phosphorus (TP) and total nitrogen (TN) make the water of the Great Salt Lake an extreme environment, which selects for a microbiota adapted to high salt concentrations.

**Table 1:** **Environmental quality parameters, nutrients, and trace metals present in Great Salt Lake**.

**Parameter**	**Average**	Standard deviation^*^
Specific conductivity (μS/cm)	188,400	11,000
Salinity (ppt)	150	5.5
pH	7.4	0.1
Dissolved oxygen (mg/l)	0.03	N/A
Total organic carbon (TOC) (mg/g)	20.8	2.6
Total nitrogen (TN) (mg/l)	4.20	0.56
NH_3_-N (mg/l)	2.29	0.8
${\text{NO}}_{3}^{-}$-N (mg/l)	0.40	0.06
Total phosphorus (TP) (mg/l)	4.47	0.27
Inorganic phosphorus (mg/l)	0.697	0.1
Orthophosphate (mg/l)	0.94	0.3
Mn (mg/kg)	0.208	N/A^*^
Mo (mg/kg)	0.174	
Zn (mg/kg)	0.077	
Fe (mg/kg)	0.036	
Ni (mg/kg)	0.031	
Se (mg/kg)	0.010	
Co (mg/kg)	0.005	
Cu (mg/kg)	0.003	
Pb (mg/kg)	0.001	

* *Except for the ICP-MS measurement, the physicochemical characteristics were measured in triplicate*.

Total organic carbon (TOC) concentration was also measured in the sediment sample, and it showed a high carbon amount of 20.8 mg.g^−1^ (2%) compared to the average TOC of 0.5% in the deep ocean (Seiter et al., 2004). Moreover, a considerable amount of ammonia (NH_3_) was measured in the sediment sample compared to the EPA aquatic life water quality criteria for ammonia (Unites States Environmental Protection Agency, Office of Water, 2013), which results in a very low total organic carbon to total nitrogen (C/N ratio) that can mobilize nitrogen in the sediment due to its excess amount.

### Prokaryote and phage metagenomes contig assembly

The high-throughput sequencing generated 15.1 million reads for each of the bacteria and phage DNA samples. Following quality and length control, 1.26 million (8.34%) and 1.27 million (8.39%) reads from the bacteria and phage samples were removed, respectively. The filtered and interleaved paired-end reads were *de novo* assembled using CLC Genomics Workbench resulting in 40,223 contigs with average length of 1,575 bp, N50 of 1,573 bp, and average coverage of 20 × for bacteria reads; and 45,689 contigs with average length of 1,595 bp, N50 value of 1,615 bp, and average coverage of 13 × for phage reads. Average coverage of the contigs was calculated by multiplying the read quantity by the average read length and dividing by the average contig length. These contigs contained 51.7 and 43.9% of the shotgun metagenomics reads in the bacterial and phage metagenomes, respectively.

Although typical prokaryotic genes (such as 16S ribosomal RNA) were never detected in the phage genomic DNA prior to sequencing, to ensure the absence of any cellular DNA in the phage sequences, genes encoding the 5S, 16S, and 23S rRNAs were identified in phage contigs. A total of 0.1% of the phage contigs were detected to have prokaryotic rRNA genes and therefore excluded from the phage contigs for further analysis.

### Prokaryote and phage contig analysis

**Figure 1:**
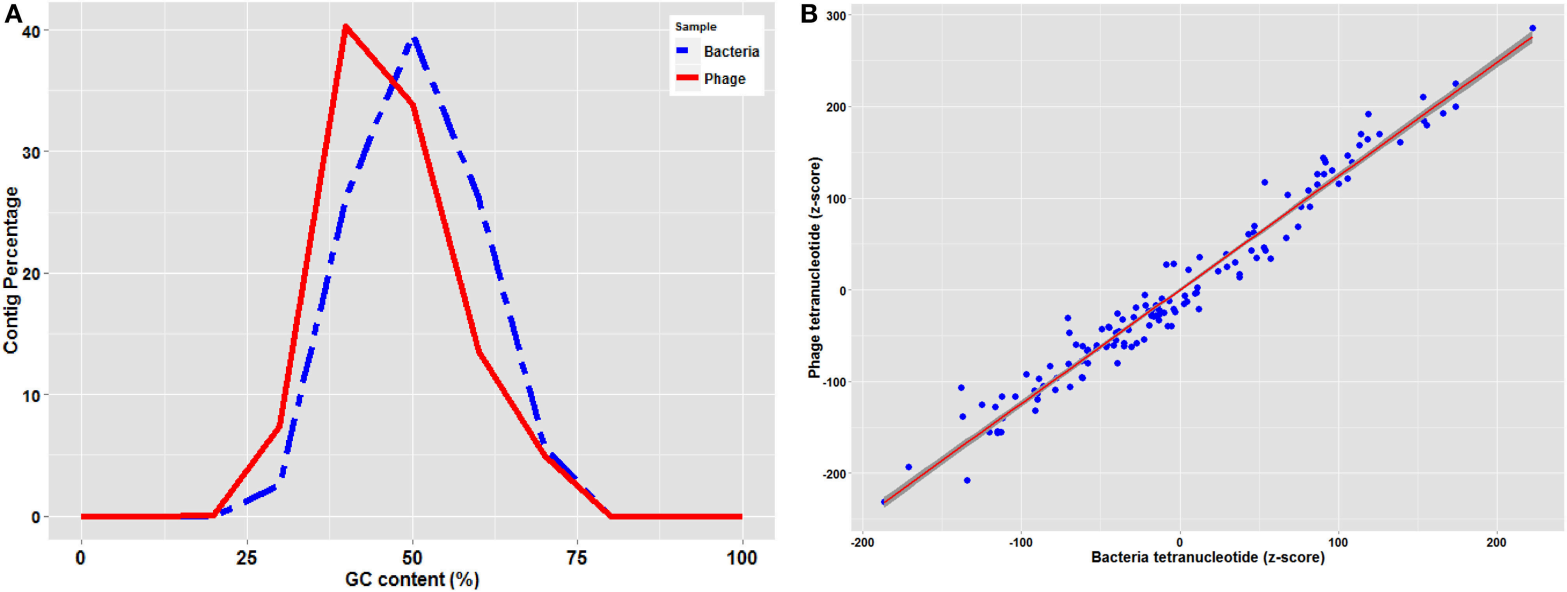
**(A)** GC content distribution of bacteria and phage contigs **(B)** Standardized (z-score) tetramer word frequencies of phage and bacteria contigs with the regression line with shaded confidence region.

Genetic elements such as phages reproduce inside prokaryote cells using the cell's replication machinery, thus most of the phages is expected to have GC content similar to that of their host. However due to horizontal gene transfer, phages tend to have an average 4% higher AT content compared to their host and therefore GC content in the phages is generally lower than their hosts (Rocha and Danchin, 2002). As shown in Figure 1A, GC content of bacterial contigs had a normal distribution of 47 ± 8% with an average of 46.4%, while phage contig GC contents were normally distributed at 43 ± 10% with an average of 42.5%.

Prophages replicate vertically with the prokaryotic chromosome and therefore are subject to “amelioration” toward the oligonucleotide usage profile of the host that they are infecting (Pride et al., 2006). Although virulent phages are not integrated as prophages and replicate independently of the bacterial chromosome, they also present a similar trend (Rocha and Danchin, 2002). Therefore, comparison of oligonucleotide usage profiles of phage and bacterial contigs can be used to predict the phage-host associations (Edwards et al., 2016). Tetranucleotide analysis plots standardized (z-score) tetramer frequencies of phage and bacterial contigs against each other and uses linear regression analysis to determine similarity. As illustrated in Figure 1B, tetranucleotide distribution plot of phage and bacterial contigs showed a consistent pattern with a regression value (r-square) of 0.98 from plotting both phage and bacterial tetranucleotide occurrences, which can provide a signal for prediction of phage-host interactions.

**Figure 2:**
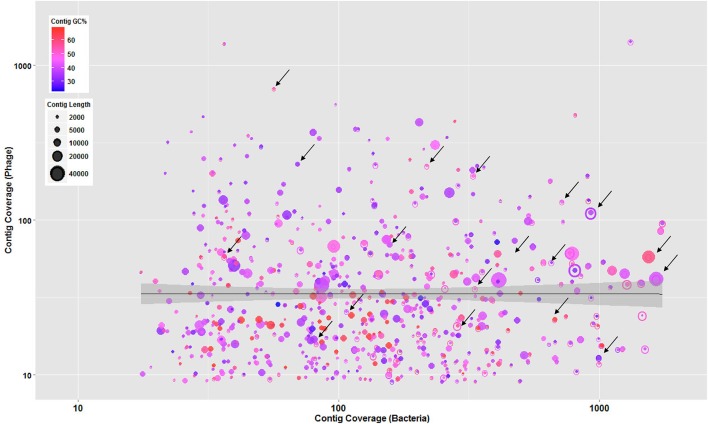
**Overlapped bacteria (open circles) and phage (solid circles) showing contigs with similar GC content and coverage suggesting that phages were well-adapted to the codon usage of their prokaryotic hosts**. All circles represent contigs, scaled by their length and colored by GC content. The regression line with shaded confidence region is shown and arrows are illustrating some of the overlapped contigs.

In addition, to understand the phage-host interaction, further criteria including contigs' GC content, length, and coverage were employed to sort the major assembly pieces. These criteria associated with phage contigs were plotted against the bacterial contigs to determine mutual scaffolds shared by bacteria and phages with similar GC content and coverage. This coverage binning approach was previously used in a study conducted by Albertsen et al. (2013) for multiple metagenomes. As shown in Figure 2, phage and bacterial contig coverage in log_10_ scale were compared along with contigs' GC content and length. Several overlapping phage and bacterial contigs with similar GC content and coverage were determined suggesting that phages were well-adapted to the codon usage of their prokaryotic hosts.

### Microbial community structure

#### Prokaryotic community structure and diversity

Microbial communities and specifically prokaryotes in natural ecosystems are involved in nutrient mobilization and regeneration, primary production, and energy fluxes. In addition, the prokaryotic populations in the sediment play key roles in biogeochemical cycles and their infection by phages can affect various nutrient cycles in which these organisms are involved. Investigating microbial diversity is therefore essential to understand the ecology and microbial interactions of Great Salt Lake.

For a better understanding of prokaryote diversity, following tBLASTx analysis against NCBI non-redundant (nr) nucleotide database with 1E-5 *e*-value cut-off, 72.3% of the contigs matched the bacterial domain, while archaea (1%), eukaryotes (6.4%), viruses (13.8%), and unassigned with no annotation (6.4%) comprised the rest of the contigs. The prokaryotic taxonomic rRNA analysis revealed that *Proteobacteria* (46.2%), *Firmicutes* (20.3%), *Bacteroidetes* (9%), *Actinobacteria* (5.3%), *Chloroflexi* (5.2%), *Cyanobacteria* (2.7%), and *Planctomycetes* (1.7%) were the most dominant bacterial phyla present in the sediment sample. Prokaryote taxonomic diversity and population found in the prokaryote contigs are presented in Figure S2, Tables S1, S4.

#### Bacteriophage community population and diversity

Since unassembled reads in viral metagenomes are often characterized by a majority of unknown sequences that have no homologs in the database (Mokili et al., 2012), assembled reads present in phage contigs were used for taxonomic characterization to facilitate their annotation. Classification of viral metagenome contigs using lowest common ancestor (LCA) analysis with the RefSeq viral database revealed *Siphoviridae* (32%), *Myoviridae* (24%), and *Podoviridae* (13%) as the most dominant viral families in the sediment sample. In addition to these dominant phage families, other phages such as unclassified dsDNA phages (6%), unclassified archaeal dsDNA virus (6%), and unclassified *Caudovirales* (3%) were among matched hits in the phage contigs. Among *Siphoviridae* viral family, unclassified lambda-like phages was the most dominant phage with 6% among the family and 2% in the entire phage community. In addition, analysis of *Myoviridae* family showed that unclassified T4-like phages and T4-like phages in the subfamily of *Tevenvirinae* was composing 7 and 8% of the *Myoviridae* family, respectively. Furthermore, *Cellulophaga* phage was among the most dominant phages in *Podoviridae* comprising 8% of the family and 1% of total phage community.

**Figure 3:**
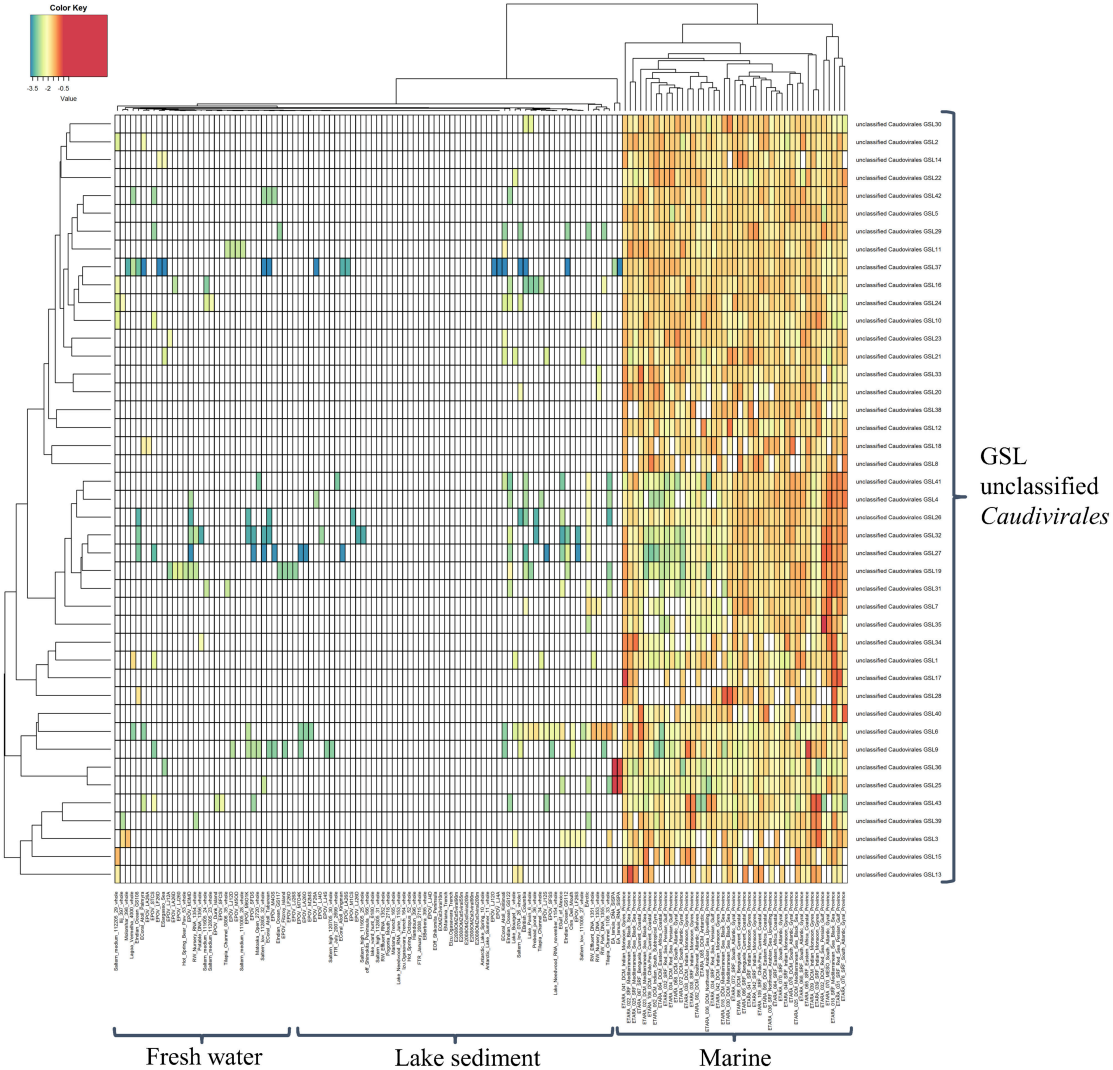
**Heatmap generated by comparison of putative complete phages from Great Salt Lake and the metagenomes of marine and freshwater lakes publicly available**. Cell color represents the relative percentage of identity (log_10_) of reads in viromes mapped to GSLs phage genome fragments.

Despite the growing knowledge for the ecological role played by bacteriophages and an increase in phage sequence information in public databases, little is known about their dynamics in natural ecosystems. It is therefore imperative to be able to classify these organisms in order to determine their dynamics. As a result, this study developed a higher resolution insight into the viral biogeography by mapping assembled phage contigs with length longer than 20 kbp as putative complete phages from the GSL to phage sequences from freshwater lakes, lake sediments, and marine ecosystems to determine the biogeographical distribution patterns of these phage genome fragments (Figure 3). In the generated heatmap, cell color represents the relative percentage of identity between GSL contigs and other publicly available phage metagenomes. From the high resolution abundance heatmap, it was interesting to observe that these novel unclassified *Caudovirales* GSL phages tend to be also abundant in marine environments as well despite Great Salt Lake being a hypersaline land-locked lake.

### Predicted gene abundance and analysis

**Figure 4:**
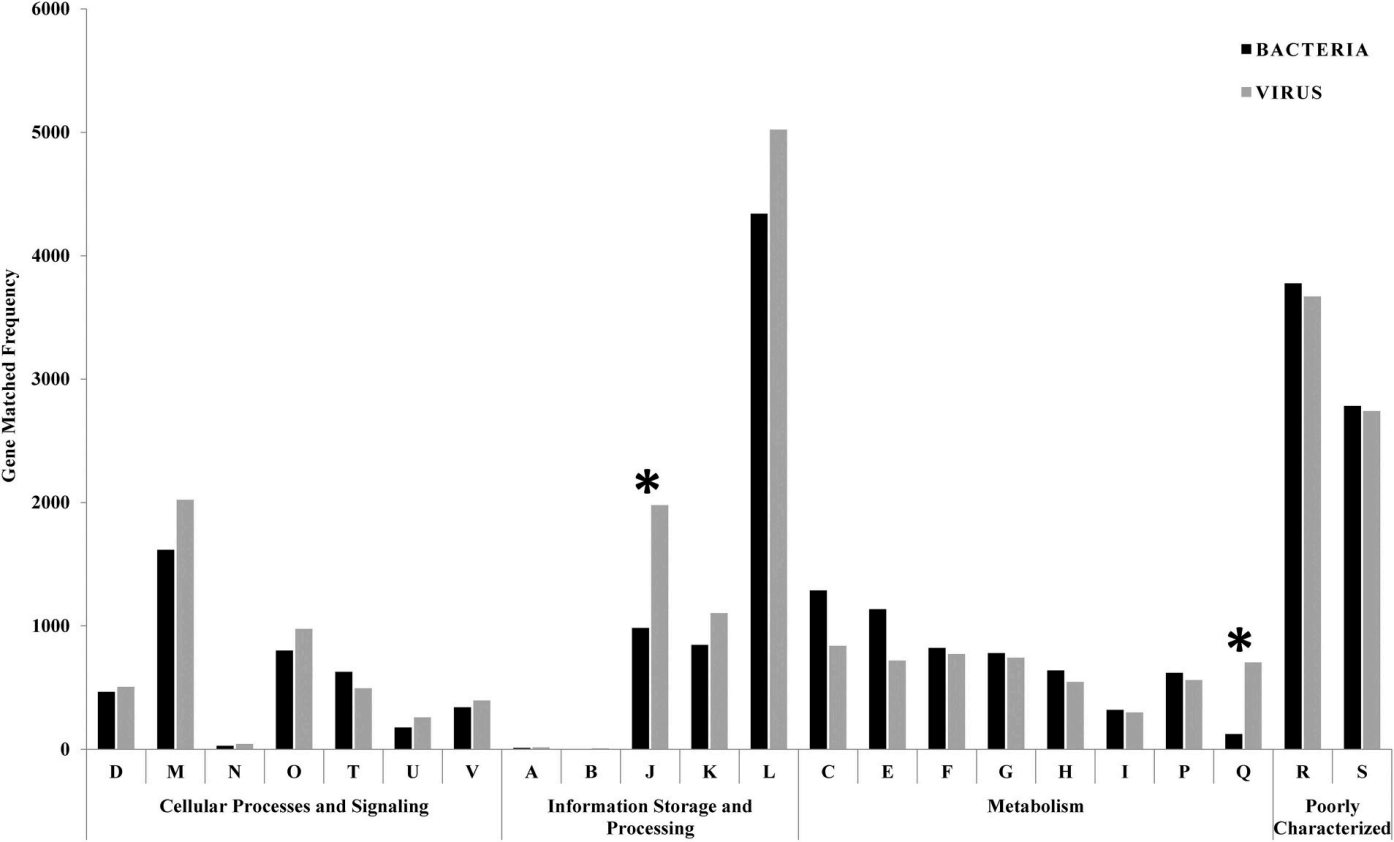
**Comparison of bacterial and viral functional gene profile classification based on COG classification scheme**. The asterisks are showing the significantly different categories (translation and secondary metabolites biosynthesis) between phage and bacterial predicted genes. Functional classes: D = cell cycle control, cell division, chromosome partitioning; M = cell wall/membrane/envelope biogenesis; *N* = cell motility; O = post-translational modification, protein turnover, and chaperones; T = signal transduction mechanisms; U = intracellular trafficking, secretion, and vesicular transport; V = defense mechanisms; A = RNA processing and modification; B = chromatin structure and dynamics; J = translation, ribosomal structure and biogenesis; K = transcription; L = replication, recombination and repair; C = energy production and conversion; E = amino acid transport and metabolism; F = nucleotide transport and metabolism; G = carbohydrate transport and metabolism; H = coenzyme transport and metabolism; I = lipid transport and metabolism; *P* = inorganic ion transport and metabolism; Q = secondary metabolites biosynthesis, transport, and catabolism; R = general function prediction only; S = function unknown.

Gene prediction and ORF identification in phage and bacterial contigs were performed to identify possible events of horizontal gene transfer between the phages and their prokaryotic hosts. GSL metagenome predicted genes were classified into one of 22 Clusters of Orthologous Groups (COG) functional categories according to best hit classification through RPS-BLAST against the COG reference database. Out of the 122,016 bacterial and 102,594 viral predicted genes, 26,319 and 24,495 could be annotated with *e*-value cut-off of 1E-3. Figure 4 shows the frequency of phage and bacterial genes in various COG classes. The category J (translation, ribosomal structure, and biogenesis) and category Q (secondary metabolites biosynthesis, transport, and catabolism), were significantly different for phage and bacterial predicted genes. Both categories have down-regulated genes (Chhabra et al., 2006) suggesting that some of the genes in these categories are responsible for the expression inhibition of host genes. In addition, genes classified in category Q have a role in response to environmental changes (Makino et al., 2003), which were significantly less available in the bacterial genes.

### Prophage identification

**Figure 5:**
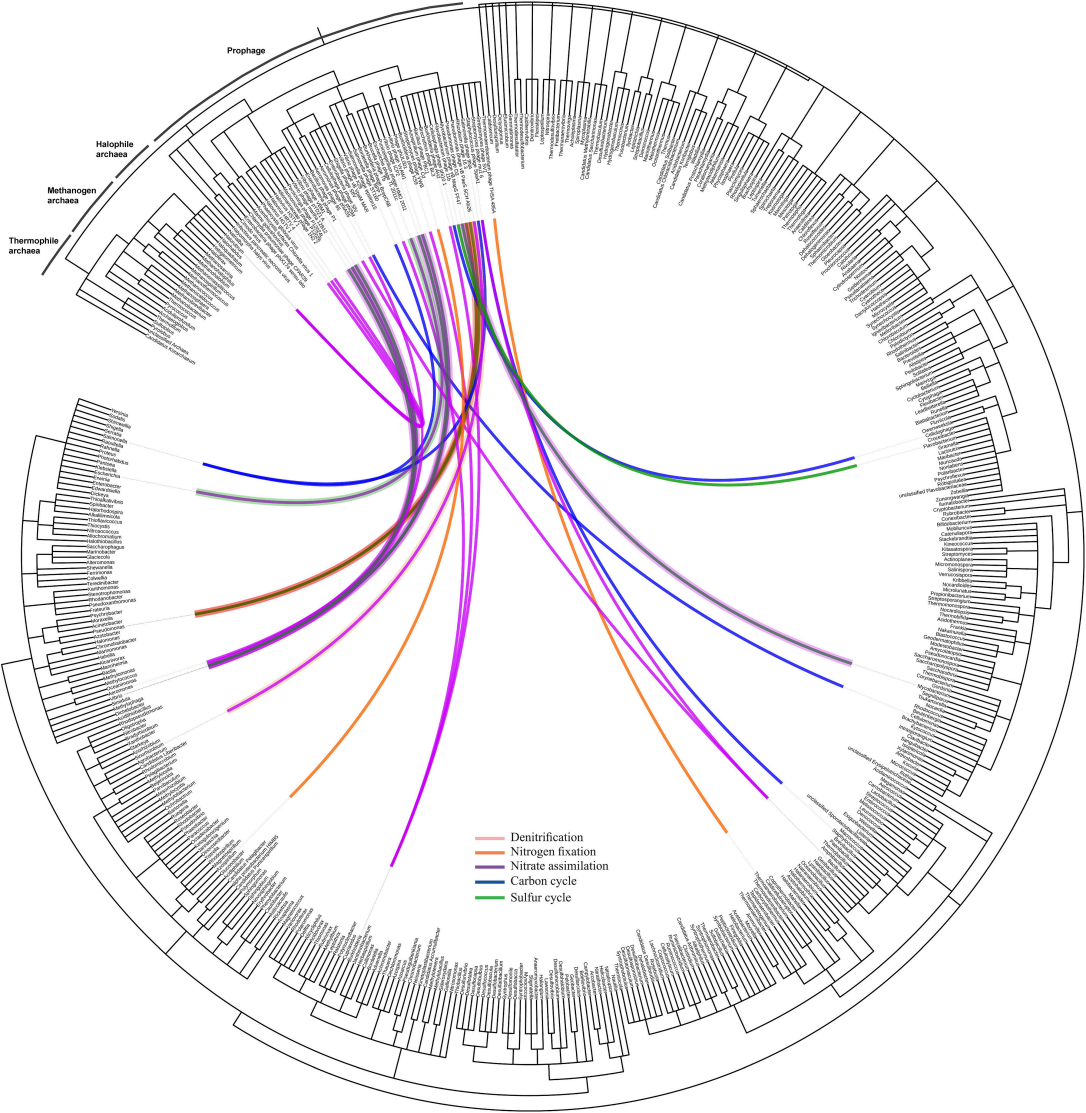
**Network of prophage and their prokaryotic hosts**. The dendrogram is based on NCBI taxonomy, showing the phage taxa that were found as integrated prophages in the bacteria contigs with respect to their hosts. The network is showing the interaction of the prophages and their prokaryotic hosts and the color of the line indicates the nutrient cycle that these prokaryotic hosts are involved in and, consequently, the susceptibility of these cycles due to presence of infecting temperate phages.

Identification of prophages in the bacterial contigs was applied to determine phage-host interactions in the natural ecosystem. Using Prophinder, a total of 69 prophages (33 intact and 36 questionable prophages) were found in the bacterial contigs. From these 69 total identified prophages, 37 prophages were joined to recognizable host DNA in the bacterial contigs and so were assigned to their prokaryotic hosts. Prokaryotic host DNAs were not found in the bacterial contigs for the other 32 putative prophages, and they were therefore excluded from further analysis. The nutrient cycling processes of the prokaryotic hosts were based on the KEGG database and were manually annotated. The taxonomy of prophages in the bacterial contigs was plotted on a circular cladogram relating the prophages and their prokaryotic hosts involved in different nutrient cycles (Figure 5), and it showed that carbon cycle was the most susceptible nutrient cycling pathway to prophage induction in the presence of environmental stresses. The numbers of different nutrient cycling pathways that can be affected by prophage induction are plotted in the Venn diagram (Figure S3) and the prophage-host interaction showing the identified prophage, their prokaryotic hosts are presented in Table S2 in detail.

### Crisprs identification

One limitation of metagenomics analyses is that when bacteria and viruses are sequenced together, it may be difficult to distinguish between the two types of sequences. Conversely, when phages are isolated and sequenced directly, it is challenging to identify the specific hosts from the viral sequence (Edwards et al., 2016). However, such information is critical for the analysis of the relationship between phage and host. In order to approach this problem, similarities between bacterial CRISPR spacers and the infecting mobile elements were analyzed to study these interactions in the natural ecosystem.

Initially, in order to associate the CRISPRs with phage sequences, the bacterial contigs as query were compared with phage contigs as database using BLASTn with an *e*-value 1E-5 cut-off. A total of 6,025 bacterial contigs with more than 70% identity in sequence with phage contigs were extracted. Using CRISPR Recognition Tool (CRT), 96 CRISPR arrays were recognized from all bacterial contigs with an average repeat length of 25 bp and spacer length of 40 bp. These identified CRISPR arrays were explored to determine targets of CRISPR RNAs using CRISPRTarget. Interestingly, the identified genome spacers showed similarity with eight of the protospacers on the marine phage metagenome from TARA database (Sunagawa et al., 2015). These were shown in the previous section to have considerable homology with the phage contigs in the GSL sample. This connection indicates that previous infections of GSL prokaryotic hosts with relatives of these phages must have occurred.

## Discussion

In this study, we used phage and bacterial metagenome sequencing to better understand the microbial interactions between the phage and their prokaryotic hosts. The nutrient concentrations in the sediment were measured to explore the microbial activities involved in different nutrient cycles. As mentioned earlier, considerable concentration of organic carbon was measured in the sediment sample. Organic carbon deposited in sediment is mainly derived from freshly deposited plant litter and decomposed forms such as humus (Kristensen and Holmer, 2001). In addition, bacterial growth and metabolic activities of methanotrophs, methanogens, and photosynthetic phytoplanktons contribute to the high concentration of TOC in sediment sample through fixation of CO_2_. As discussed on prokaryotic community structure, our metagenomic analysis shows the presence of halophilic and halotolerant methane-producing and methanotroph bacteria in the GSL sediments supporting the presence of such bacterial carbon sources in the sediment. Furthermore, high concentrations of nutrients can increase phytoplankton growth and consequently increase of debris sedimentation. As a consequence of the increased productivity that results in oxygen depletion, sediments in the anoxic environment of the lake will contain a larger amount of organic matter (Lazar et al., 2012). The nitrifiers including *Nitrospirae, Nitrosomonas*, and *Nitrobacter*, and denitrifiers including *Bacillus, Pseudomonas, Clostridium, Halomonas*, and *Rhodobacter* were among the most abundant phyla found in the prokaryotic contigs, which considerably affect the nitrogen cycle. In addition, ammonia concentrations following the appearance of brine fly larvae and brine shrimp in the GSL in early summer (Wurtsbaugh, 1992) when algal populations decline due to grazing and nitrogenous wastes from the invertebrates increase. Furthermore, ammonium (${\text{NH}}_{4}^{+}$) can arise from decomposition of organic matter in the water column or from diffusion of ammonium from the anaerobic sediment layer into the water column (Chowdhury and Bakri, 2006). Furthermore, presence of nitrogen fixing bacterial family such as *Beijerinckiaceae* as well as dissimilatory nitrate reduction to ammonium (DNRA) bacterial family such as *Geobacteraceae* in the analyzed bacterial metagenome were contributing to increase the level of ammonia in the sediment sample. The levels of trace metals observed at the deep brine layer suggest that the sediment may be a hotspot for nutrient cycles. Many enzymatic pathways of the nitrogen, carbon, and sulfur cycles require cofactors which contain iron or molybdenum. In particular, iron is a necessary requirement and an integral component of many enzymes involved in photosynthesis, electron transport, and nutrient acquisition (Geider and La Roche, 1994). Furthermore, molybdenum is a required component of enzymes involved in nitrogen cycling pathways such as nitrate assimilation and nitrogen fixation (Glass et al., 2015). For instance, nitrogenase, a critical multi-component enzyme in nitrogen fixation typically consists of iron and molybdenum-iron containing subunits (Howard and Rees, 1996). As discussed in the following gene analysis, these critical enzymes involved in various nutrient cycles were found in both prokaryote and phage metagenomes, which suggests a phage-mediated gene transfer process.

More than 450 different genera were classified in the sediment sample, while the most abundant genera, in order of abundance, are *Desulfococcus, Halanarobium, Desulfovibrio, Desulfatibacillum*, and *Streptomyces*, which belong to taxa involved in various biogeochemical cycles. *Desulfococcus* plays an important role in cycles of sulfur compounds in sea water (Das et al., 2006). *Halanaerobium* is an obligatory anaerobic halophile, and several strains of *Halanaerobium* such as *H. praevalens* were previously isolated from GSL and showed complex organic matter fermentation and production of intermediary metabolites for other trophic groups such as sulfate-reducing and methanogenic bacteria (Ivanova et al., 2011). Presence of *Desulfovibrio* and *Desulfatibacillum*, as sulfate-reducing anaerobic bacterial genera, was consistent with the anoxic condition of the brine layer sediment. *Streptomyces* is an important organism in carbon recycling that has a crucial role in the environment since it carries out a broad range of metabolic processes such as degradation of insoluble biological material including lignocellulose and chitin (Bentley et al., 2002). *Proteobacteria* of the genus *Rhodobacter* are metabolically versatile, capable of aerobic and anaerobic respiration, and anoxygenic photosynthesis when grown anaerobically in the light (Han et al., 2004).

*Caudovirales* are tailed bacteriophages, which are found to dominate in marine and other aquatic environments (Weinbauer, 2004; Winter et al., 2014). In a recent study, Antunes et al. (2015) studied viral communities in deep sea anoxic brine of Red Sea and found *Siphoviridae* and *Myoviridae* to be dominant viral family members yet appearing distinct from sample to sample. We found that *Siphoviridae* (32%) in our analysis was the most dominant viral family indicating that perhaps high salt concentration does not put a selective pressure on viral communities. However, these results should be further confirmed with comprehensive analysis involving time series metagenomics of samples collected from different salinity gradients. As mentioned in the results section, we found lambda-like, T4-like, and *Cellulophaga* phages as some of the most abundant genera among various phage families. In a metagenomic study by Ray et al. (2012) at the Arctic Mid-Ocean Ridge, high abundance of lambda-like phage sequences in both hydrothermal plume and surrounding seawater was also found showing significant ecological role of this phage genus in various ecosystems. Furthermore, T4-like phages were previously identified in significant number of sequences in metagenomic study on two freshwater viral communities (Roux et al., 2012). This suggests that viral communities in the hypersaline ecosystem studied in our research does not appear genetically distinct from other aquatic ecosystems. In addition, a previous study conducted with Holmfeldt et al. (2013) revealed that *Cellulophaga* phage genus was widespread and ubiquitous fraction among marine viral diversity.

For the COG analysis, category A is attributed to RNA processing and was measured in negligible match hits (10 and 15 match hits for bacterial and viral genes, respectively). As chromatin forms chromosomes within the nucleus of eukaryotic cells, category B belongs only to eukaryotes and was detected in minor match hits (8 and 5 match his in bacterial and viral genes, respectively) in our samples. Based on Fisher's exact test, there were significant differences in the functional gene profiles between COG gene categories (*p* < 0.05). There was an over-representation of genes classified into the “replication, recombination, and repair” category (category L) with 4,341 and 5,022 match hits for bacterial and viral genes, respectively. The most frequent gene among all bacterial predicted genes (occurrence = 416), and the second most frequent gene in the viral predicted genes (occurrence = 354) was related to replicative DNA helicase (COG0305), an enzyme that participates in initiation and elongation during chromosome replication with unwinding DNA and exhibiting DNA-dependent ATPase activity. In addition, bacterial (314 match hits) and viral (340 match hits) metagenomes showed a high representation of the DNA modification methylase (COG0863) gene, which is a part of the restriction-modification systems and responsible for producing a species-characteristic methylation pattern that can be used to protect the bacteria from foreign DNA, such as that borne by bacteriophages.

The COG analysis allowed summarizing the functional gene data and showing the differences in functional trends of bacteria and phage samples. In addition to COG analysis, the predicted genes were also categorized into SEED Subsystems through MG-RAST pipeline. The common proteins in bacterial contigs which were also found in phage contigs were extracted (Table S3). The presence of these enzymes in the bacteria and phage contigs suggests that phage-mediated gene transfer influences environmental processes.

## Conclusions

The Great Salt Lake is one of the most unique hypersaline environments on the planet and has a high diversity in bacteria and bacteriophage species, and many of the bacteria are involved in various nutrient cycles. Metagenomics analyses of bacteria and phage sequences revealed the relationship between the phage and their prokaryotic hosts through their GC content, contig coverage, scaffold length, and tetranucleotide frequency. Furthermore, the presence of prophages and their interaction with the prokaryotic host showed the susceptibility of Great Salt Lake environment to possible prophage induction under different environmental stress factors. In addition, gene prediction in the bacteria and phage contigs showed shared functional genes in the samples, which suggests that gene transfer between bacteria that is meditated by phage transduction and phage-like gene transfer agents (Lang and Beatty, 2007; Stanton, 2007). Lastly, identification of CRISPRs in the bacterial contigs confirms prevalence of previous infections among the prokaryotic community by phages similar to those in marine environments. The phage-host interactions in hypersaline ecosystems are complex and our study gives an enhanced glimpse of phage and prokaryote abundance and diversity as well as their interactions in the hypersaline environment of Great Salt Lake. We also gained interesting information on prophages and how they can affect the prokaryote diversity and nutrient cycling, which are valuable pieces of information for environmental microbiology and ecology.

## Author contributions

AM and AB designed and performed laboratory analyses, analyzed data, and contributed to write the manuscript. FC, BD, and SC helped in analyzing and interpreting the data and contributed to writing the manuscript. RG is the primary investigator of this research and contributed in designing the experiments and final approval of the version to be published.

### Conflict of interest statement

The authors declare that the research was conducted in the absence of any commercial or financial relationships that could be construed as a potential conflict of interest.

## References

[B1] Albertsen M. Hugenholtz P. Skarshewski A. Nielsen K. L. Tyson G. W. Nielsen P. H. (2013). Genome sequences of rare, uncultured bacteria obtained by differential coverage binning of multiple metagenomes. Nature Biotechnol. 31, 533–538. 10.1038/nbt.2579 23707974

[B2] Altschul S. F. Gish W. Miller W. Myers E. W. Lipman D. J. (1990). Basic local alignment search tool. J. Mol. Biol. 215, 403–410. 10.1016/S0022-2836(05)80360-2 2231712

[B3] Anderson R. E. Brazelton W. J. Baross J. A. (2011). Using CRISPRs as a metagenomic tool to identify microbial hosts of a diffuse flow hydrothermal vent viral assemblage. FEMS Microbiol. Ecol. 77, 120–133. 10.1111/j.1574-6941.2011.01090.x 21410492

[B4] Angly F. E. Felts B. Breitbart M. Salamon P. Edwards R. A. Carlson C.. (2006). The marine viromes of four oceanic regions. PLoS Biol. 4:e368. 10.1371/journal.pbio.0040368 17090214 PMC1634881

[B5] Antunes A. Alam I. Simões M. F. Daniels C. Ferreira A. J. S. Siam R.. (2015). First insights into the viral communities of the Deep-sea Anoxic brines of the Red Sea. Genomics Proteomics Bioinformatics 13, 304–309. 10.1016/j.gpb.2015.06.004 26529193 PMC4678784

[B6] Bahr M. Crump B. C. Klepac-Ceraj V. Teske A. Sogin M. L. Hobbie J. E. (2005). Molecular characterization of sulfate-reducing bacteria in a New England salt marsh. Environ. Microbiol. 7, 1175–1185. 10.1111/j.1462-2920.2005.00796.x 16011754

[B7] Bentley S. D. Chater K. F. Cerdeño-Tárraga A. M. Challis G. L. Thomson N. R. James K. D.. (2002). Complete genome sequence of the model actinomycete Streptomyces coelicolor A3 (2). Nature 417, 141–147. 10.1038/417141a 12000953

[B8] Bhattacharjee A. S. Choi J. Motlagh A. M. Mukherji S. T. Goel R. (2015). Bacteriophage therapy for membrane biofouling in membrane bioreactors and antibiotic-resistant bacterial biofilms. Biotechnol. Bioeng. 112, 1644–1654. 10.1002/bit.25574 25728819

[B9] Biswas A. Gagnon J. N. Brouns S. J. Fineran P. C. Brown C. M. (2013). CRISPRTarget: Bioinformatic prediction and analysis of crRNA targets. RNA Biol. 10, 817–827. 10.4161/rna.24046 23492433 PMC3737339

[B10] Bland C. Ramsey T. L. Sabree F. Lowe M. Brown K. Kyrpides N. C.. (2007). CRISPR recognition tool (CRT): a tool for automatic detection of clustered regularly interspaced palindromic repeats. BMC Bioinformatics 8:209. 10.1186/1471-2105-8-209 17577412 PMC1924867

[B11] Casjens S. (2003). Prophages and bacterial genomics: what have we learned so far?. Mol. Microbiol. 49, 277–300. 10.1046/j.1365-2958.2003.03580.x 12886937

[B12] Chavan P. V. Dennett K. E. Marchand E. A. Gustin M. S. (2007). Evaluation of small-scale constructed wetland for water quality and Hg transformation. J. Hazard. Mater. 149, 543–547. 10.1016/j.jhazmat.2007.06.077 17693019

[B13] Chen F. Suttle C. A. Short S. M. (1996). Genetic diversity in marine algal virus communities as revealed by sequence analysis of DNA polymerase genes. Appl. Environ. Microbiol. 62, 2869–2874. 8702280 10.1128/aem.62.8.2869-2874.1996 PMC168073

[B14] Chhabra S. R. He Q. Huang K. H. Gaucher S. P. Alm E. J. He Z.. (2006). Global analysis of heat shock response in Desulfovibrio vulgaris Hildenborough. J. Bacteriol. 188, 1817–1828. 10.1128/JB.188.5.1817-1828.2006 16484192 PMC1426554

[B15] Chowdhury M. Bakri D. A. (2006). Diffusive nutrient flux at the sediment—water interface in Suma Park Reservoir, Australia. Hydrol. Sci. J. 51, 144–156. 10.1623/hysj.51.1.144

[B16] Dagan T. Artzy-Randrup Y. Martin W. (2008). Modular networks and cumulative impact of lateral transfer in prokaryote genome evolution. Proc. Natl Acad. Sci. U.S.A. 105, 10039–10044. 10.1073/pnas.0800679105 18632554 PMC2474566

[B17] Das S. Lyla P. S. Khan S. A. (2006). Marine microbial diversity and ecology: importance and future perspectives. Curr. Sci. 90, 1325–1335.

[B18] Del Casale A. Flanagan P. V. Larkin M. J. Allen C. C. R. Kulakov L. A. (2011). Analysis of transduction in wastewater bacterial populations by targeting the phage-derived 16S rRNA gene sequences. FEMS Microbiol. Ecol. 76, 100–108. 10.1111/j.1574-6941.2010.01034.x 21223328

[B19] Diemer G. S. Stedman K. M. (2012). A novel virus genome discovered in an extreme environment suggests recombination between unrelated groups of RNA and DNA viruses. Biol. Direct. 7:13. 10.1186/1745-6150-7-13 22515485 PMC3372434

[B20] Dutilh B. E. Thompson C. C. Vicente A. C. Marin M. A. Lee C. Silva G. G.. (2014). Comparative genomics of 274 Vibrio cholerae genomes reveals mobile functions structuring three niche dimensions. BMC Genomics 15:654. 10.1186/1471-2164-15-654 25096633 PMC4141962

[B21] Edwards R. McNair K. Wellington-Oquri M. Faust K. Raes J. Dutilh B. E. (2016). Computational approaches to predict bacteriophage-host relationships. FEMS Micriobiol. Rev. 40, 258–272. 10.1093/femsre/fuv048 26657537 PMC5831537

[B22] Faulwetter J. L. Burr M. D. Parker A. E. Stein O. R. Camper A. K. (2013). Influence of season and plant species on the abundance and diversity of sulfate reducing bacteria and ammonia oxidizing bacteria in constructed wetland microcosms. Microb. Ecol. 65, 111–127. 10.1007/s00248-012-0114-y 22961363

[B23] Finn R. D. Clements J. Eddy S. R. (2011). HMMER web server: interactive sequence similarity searching. Nucleic Acids Res. 39 (suppl_2), W29–W37. 10.1093/nar/gkr367 21593126 PMC3125773

[B24] Fish J. A. Chai B. Wang Q. Sun Y. Brown C. T. Tiedje J. M.. (2013). FunGene: the functional gene pipeline and repository. Front. Microbiol. 4:291. 10.3389/fmicb.2013.00291 24101916 PMC3787254

[B25] Fortin D. Goulet R. Roy M. (2000). Seasonal cycling of Fe and S in a constructed wetland: the role of sulfate-reducing bacteria. Geomicrobiol. J. 17, 221–235. 10.1080/01490450050121189

[B26] Geider R. J. La Roche J. (1994). The role of iron in phytoplankton photosynthesis, and the potential for iron-limitation of primary productivity in the sea. Photosyn. Res. 39, 275–301. 10.1007/BF00014588 24311126

[B27] Glass J. B. Axler R. P. Chandra S. Goldman C. R. (2015). Molybdenum limitation of microbial nitrogen assimilation in aquatic ecosystems and pure cultures. Front. Microbiol. 3:331. 10.3389/fmicb.2012.00331 22993512 PMC3440940

[B28] Gough H. L. Stahl D. A. (2010). Microbial community structures in anoxic freshwater lake sediment along a metal contamination gradient. ISME J. 5, 543–558. 10.1038/ismej.2010.132 20811473 PMC3105716

[B29] Han Y. Braatsch S. Osterloh L. Klug G. (2004). A eukaryotic BLUF domain mediates light-dependent gene expression in the purple bacterium *Rhodobacter sphaeroides* 2.4. 1. Proc. Natl. Acad. Sci. U.S.A. 101, 12306–12311. 10.1073/pnas.0403547101 15292515 PMC514474

[B30] Heidelberg J. F. Nelson W. C. Schoenfeld T. Bhaya D. (2009). Germ warfare in a microbial mat community: CRISPRs provide insights into the co-evolution of host and viral genomes. PLoS ONE 4:e4169. 10.1371/journal.pone.0004169 19132092 PMC2612747

[B31] Holmfeldt K. Solonenko N. Shah M. Corrier K. Riemann L. VerBerkmoes N. C.. (2013). Twelve previously unknown phage genera are ubiquitous in global oceans. Proc. Natl. Acad. Sci. U.S.A. 110, 12798–12803. 10.1073/pnas.1305956110 23858439 PMC3732932

[B32] Horvath P. Barrangou R. (2010). CRISPR/Cas, the immune system of bacteria and archaea. Science 327, 167–170. 10.1126/science.1179555 20056882

[B33] Howard J. B. Rees D. C. (1996). Structural basis of biological nitrogen fixation. Chem. Rev. 96, 2965–2982. 10.1021/cr9500545 11848848

[B34] Huson D. H. Auch A. F. Qi J. Schuster S. C. (2007). MEGAN analysis of metagenomic data. Genome Res. 17, 377–386. 10.1101/gr.5969107 17255551 PMC1800929

[B35] Ivanova N. Sikorski J. Chertkov O. Nolan M. Lucas S. Hammon N.. (2011). Complete genome sequence of the extremely halophilic Halanaerobium praevalens type strain (GSLT). Stand. Genomic Sci. 4, 312. 10.4056/sigs.1824509 21886858 PMC3156398

[B36] Jackson E. F. Jackson C. R. (2008). Viruses in wetland ecosystems. Freshw. Biol. 53, 1214–1227. 10.1111/j.1365-2427.2007.01929.x

[B37] Jasser I. Kostrzewska-Szlakowska I. Ejsmont-Karabin J. Kalinowska K. Węgleńska T. (2009). Autotrophic versus heterotrophic production and components of trophic chain in humic lakes: the role of microbial communities. Polish J. Ecol. 57, 423–439.

[B38] Jiang S. C. Paul J. H. (1998). Gene transfer by transduction in the marine environment. Appl. Environ. Microbiol. 64, 2780–2787. 9687430 10.1128/aem.64.8.2780-2787.1998 PMC106772

[B39] Kanehisa M. Araki M. Goto S. Hattori M. Hirakawa M. Itoh M.. (2008). KEGG for linking genomes to life and the environment. Nucleic Acids Res. 36, D480–D484. 10.1093/nar/gkm882 18077471 PMC2238879

[B40] Kenzaka T. Tani K. Nasu M. (2010). High-frequency phage-mediated gene transfer in freshwater environments determined at single-cell level. ISME J. 4, 648–659. 10.1038/ismej.2009.145 20090786

[B41] Kerr Y. H. Waldteufel P. Wigneron J. P. Font J. Berger M. (2003). The soil moisture and ocean salinity mission, in Geoscience and Remote Sensing Symposium, 2003. IGARSS'03. Proceedings 2003 IEEE International, Vol. 1, (IEEE), 1–3. 10.1109/IGARSS.2003.1293658

[B42] Kidambi S. P. Ripp S. Miller R. V. (1994). Evidence for phage-mediated gene transfer among *Pseudomonas aeruginosa* strains on the phylloplane. Appl. Environ. Microbiol. 60, 496–500. 8135513 10.1128/aem.60.2.496-500.1994 PMC201339

[B43] Kim K. H. Chang H. W. Nam Y. D. Roh S. W. Kim M. S. Sung Y.. (2008). Amplification of uncultured single-stranded DNA viruses from rice paddy soil. Appl. Environ. Microbiol. 74, 5975–5985. 10.1128/AEM.01275-08 18708511 PMC2565953

[B44] Kim O. S. Imhoff J. F. Witzel K. P. Junier P. (2011). Distribution of denitrifying bacterial communities in the stratified water column and sediment–water interface in two freshwater lakes and the Baltic Sea. Aquatic Ecol. 45, 99–112. 10.1007/s10452-010-9335-7

[B45] Knowles B. Silveira C. B. Bailey B. A. Barott K. Cantu V. A. Cobián-Güemes A. G.. (2016). Lytic to temperate switching of viral communities. Nature 531, 466–470. 10.1038/nature17193 26982729

[B46] Köchl S. Niederstätter H. Parson W. (2005). DNA extraction and quantitation of forensic samples using the phenol-chloroform method and real-time PCR. Forensic DNA Typing Protoc. 13–29. 15570097 10.1385/1-59259-867-6:013

[B47] Koonin E. V. Makarova A. S. Aravind L. (2001). Horizontal gene transfer in Prokaryotes. Quantification Classification. Annu. Rev. Microbiol. 55, 709–742. 10.1146/annurev.micro.55.1.709 11544372 PMC4781227

[B48] Kouki S. M'hiri F. Saidi N. Belaïd S. Hassen A. (2009). Performances of a constructed wetland treating domestic wastewaters during a macrophytes life cycle. Desalination 246, 452–467. 10.1016/j.desal.2008.03.067

[B49] Kristensen E. Holmer M. (2001). Decomposition of plant materials in marine sediment exposed to different electron acceptors (O_2_, ${\text{NO}}_{3}^{-}$, and ${\text{SO}}_{4}^{2-}$), with emphasis on substrate origin, degradation kinetics, and the role of bioturbation. Geochim. Cosmochim. Acta 65, 419–433. 10.1016/S0016-7037(00)00532-9

[B50] Kunin V. He S. Warnecke F. Peterson S. B. Martin H. G. Haynes M.. (2008). A bacterial metapopulation adapts locally to phage predation despite global dispersal. Genome Res. 18, 293–297. 10.1101/gr.6835308 18077539 PMC2203627

[B51] Lang A. S. Beatty J. T. (2007). Importance of widespread gene transfer agent genes in α-proteobacteria. Trends Microbiol. 15, 54–62. 10.1016/j.tim.2006.12.001 17184993

[B52] Langmead B. Salzberg S. L. (2012). Fast gapped-read alignment with Bowtie 2. Nat. Methods 9, 357–359. 10.1038/nmeth.1923 22388286 PMC3322381

[B53] Lasken R. S. Stockwell T. B. (2007). Mechanism of chimera formation during the multiple displacement amplification reaction. BMC Biotechnol. 7:19. 10.1186/1472-6750-7-19 17430586 PMC1855051

[B54] Lazar L. Gomoiu M. T. Boicenco L. Vasiliu D. (2012). Total organic carbon (Toc) of the surface layer sediments covering the Seafloor of the Romanian Black Sea Coast. GeoEcoMarina 18, 121 10.5281/zenodo.56875

[B55] Leplae R. Hebrant A. Wodak S. J. Toussaint A. (2004). ACLAME: a CLAssification of Mobile genetic Elements. Nucleic Acids Res. 32, D45–D49. 10.1093/nar/gkh084 14681355 PMC308818

[B56] Letunic I. Bork P. (2007). Interactive Tree Of Life (iTOL): an online tool for phylogenetic tree display and annotation. Bioinformatics 23, 127–128. 10.1093/bioinformatics/btl529 17050570

[B57] Li C. H. Wong Y. S. Tam N. F. Y. (2010). Anaerobic biodegradation of polycyclic aromatic hydrocarbons with amendment of iron (III) in mangrove sediment slurry. Bioresour. Technol. 101, 8083–8092. 10.1016/j.biortech.2010.06.005 20594830

[B58] Lima-Mendez G. Van Helden J. Toussaint A. Leplae R. (2008). Prophinder: a computational tool for prophage prediction in prokaryotic genomes. Bioinformatics 24, 863–865. 10.1093/bioinformatics/btn043 18238785

[B59] Makino K. Oshima K. Kurokawa K. Yokoyama K. Uda T. Tagomori K.. (2003). Genome sequence of Vibrio parahaemolyticus: a pathogenic mechanism distinct from that of V cholerae. Lancet 361, 743–749. 10.1016/S0140-6736(03)12659-1 12620739

[B60] McDaniel L. Paul J. H. (2005). Effect of nutrient addition and environmental factors on prophage induction in natural populations of marine Synechococcus species. Appl. Environ. Microbiol. 71, 842–850. 10.1128/AEM.71.2.842-850.2005 15691939 PMC546667

[B61] Meyer F. Paarmann D. D'Souza M. Olson R. Glass E. M. Kubal M.. (2008). The metagenomics RAST server–a public resource for the automatic phylogenetic and functional analysis of metagenomes. BMC Bioinformatics 9:386. 10.1186/1471-2105-9-386 18803844 PMC2563014

[B62] Middelboe M. (2008). Microbial Disease in the Sea: Effects of Viruses on Carbon and Nutrient Cycling. Princeton, NJ: Princeton University Press.

[B63] Mitchell C. P. Branfireun B. A. Kolka R. K. (2009). Methylmercury dynamics at the upland-peatland interface: Topographic and hydrogeochemical controls. Water Resour. Res. 45, W02406 10.1029/2008WR006832

[B64] Mokili J. L. Rohwer F. Dutilh B. E. (2012). Metagenomics and future perspectives in virus discovery. Curr. Opin. Virol. 2, 63–77. 10.1016/j.coviro.2011.12.004 22440968 PMC7102772

[B65] Motlagh A. M. Bhattacharjee A. S. Goel R. (2015). Microbiological study of bacteriophage induction in the presence of chemical stress factors in enhanced biological phosphorus removal (EBPR). Water Res. 81, 1–14. 10.1016/j.watres.2015.04.023 26024959

[B66] Motlagh A. M. Bhattacharjee A. S. Goel R. (2016). Biofilm control with natural and genetically-modified phages. World J. Microbiol. Biotechnol. 32, 1–10. 10.1007/s11274-016-2009-4 26931607

[B67] Motlagh A. M. Goel R. (2014). Sustainability of Activated Sludge Processes. Water Reclamation and Sustainability. Waltham, MA: Elsevier Science Ltd.

[B68] Olendzenski L. Gogarten J. P. (2009). Evolution of genes and organisms: the tree/web of life in light of horizontal gene transfer. Ann. N. Y. Acad. Sci. 1178, 137–145. 10.1111/j.1749-6632.2009.04998.x 19845634

[B69] Overbeek R. Begley T. Butler R. M. Choudhuri J. V. Chuang H. Y. Cohoon M.. (2005). The subsystems approach to genome annotation and its use in the project to annotate 1000 genomes. Nucleic Acids Res. 33, 5691–5702. 10.1093/nar/gki866 16214803 PMC1251668

[B70] Overbeek R. Olson R. Pusch G. D. Olsen G. J. Davis J. J. Disz T.. (2014). The SEED and the Rapid Annotation of microbial genomes using Subsystems Technology (RAST). Nucleic Acids Res. 42, D206–D214. 10.1093/nar/gkt1226 24293654 PMC3965101

[B71] Paul J. H. (2008). Prophages in marine bacteria: dangerous molecular time bombs or the key to survival in the seas? ISME J. 2, 579–589. 10.1038/ismej.2008.35 18521076

[B72] Pride D. T. Wassenaar T. M. Ghose C. Blaser M. J. (2006). Evidence of host-virus co-evolution in tetranucleotide usage patterns of bacteriophages and eukaryotic viruses. BMC Genomics 7:8. 10.1186/1471-2164-7-8 16417644 PMC1360066

[B73] Puigbó P. Wolf Y. I. Koonin E. V. (2010). The tree and net components of prokaryote evolution. Genome Biol. Evol. 2, 745–756. 10.1093/gbe/evq062 20889655 PMC2997564

[B74] Rafferty J. P. (2011). Lakes and Wetlands. P. 112. New York, NY: The Rosen Publishing Group.

[B75] Ramasamy E. V. Toms A. Shylesh C. M. S. Jayasooryan K. K. Mahesh M. (2012). Mercury fractionation in the sediments of Vembanad wetland, west coast of India. Environ. Geochem. Health 34, 575–586. 10.1007/s10653-012-9457-z 22565490

[B76] Ray J. Dondrup M. Modha S. Steen I. H. Sandaa R. A. Clokie M. (2012). Finding a needle in the virus metagenome haystack-micro-metagenome analysis captures a snapshot of the diversity of a bacteriophage armoire. PLoS ONE 7:e34238. 10.1371/journal.pone.0034238 22509283 PMC3324506

[B77] Richter M. Rossello-Mora R. (2009). Shifting the genomic gold standard for the prokaryotic species definition. Proc. Natl. Acad. Sci. U.S.A. 106, 19126–19131. 10.1073/pnas.0906412106 19855009 PMC2776425

[B78] Rocha E. P. Danchin A. (2002). Base composition bias might result from competition for metabolic resources. Trends Genetics 18, 291–294. 10.1016/S0168-9525(02)02690-2 12044357

[B79] Rohwer F. Edwards R. (2002). The phage proteomic tree: a genome-based taxonomy for phage. J. Bacteriol. 184, 4529–4535. 10.1128/JB.184.16.4529-4535.2002 12142423 PMC135240

[B80] Rohwer F. Thurber R. V. (2009). Viruses manipulate the marine environment. Nature 459, 207–212. 10.1038/nature08060 19444207

[B81] Roux S. Enault F. Robin A. Ravet V. Personnic S. Theil S.. (2012). Assessing the diversity and specificity of two freshwater viral communities through metagenomics. PLoS ONE 7:e33641. 10.1371/journal.pone.0033641 22432038 PMC3303852

[B82] Roux S. Faubladier M. Mahul A. Paulhe N. Bernard A. Debroas D.. (2011). Metavir: a web server dedicated to virome analysis. Bioinformatics 27, 3074–3075. 10.1093/bioinformatics/btr519 21911332

[B83] Sano E. Carlson S. Wegley L. Rohwer F. (2004). Movement of viruses between biomes. Appl. Environ. Microbiol. 70, 5842–5846. 10.1128/AEM.70.10.5842-5846.2004 15466522 PMC522096

[B84] Seiter K. Hensen C. Schroter J. Zabel M. (2004). Organic carbon content in surface sediments-defining regional provinces. Deep Sea Res. Part I. 51, 2001–2026. 10.1016/j.dsr.2004.06.014

[B85] Short C. M. Suttle C. A. (2005). Nearly identical bacteriophage structural gene sequences are widely distributed in both marine and freshwater environments. Appl. Environ. Microbiol. 71, 480–486. 10.1128/AEM.71.1.480-486.2005 15640224 PMC544240

[B86] Short S. M. Suttle C. A. (1999). Use of the polymerase chain reaction and denaturing gradient gel electrophoresis to study diversity in natural virus communities. Hydrobiologia 401, 19–32. 10.1023/A:1003711115967

[B87] Sorokin D. Y. Zacharova E. E. Pimenov N. V. Tourova T. P. Panteleeva A. N. Muyzer G. (2012). Sulfidogenesis in hypersaline chloride–sulfate lakes of Kulunda Steppe (Altai, Russia). FEMS Microbiol. Ecol. 79, 445–453. 10.1111/j.1574-6941.2011.01228.x 22092787

[B88] Stanton T. B. (2007). Prophage-like gene transfer agents—novel mechanisms of gene exchange for Methanococcus, Desulfovibrio, Brachyspira, and Rhodobacter species. Anaerobe 13, 43–49. 10.1016/j.anaerobe.2007.03.004 17513139

[B89] Stephens D. (1998). Salinity-induced changes in the aquatic ecosystem of Great Salt Lake, Utah. Mod. Anc. Lake Syst. New Probl. Perspect. 1998, 1–8.

[B90] Stern A. Sorek R. (2011). The phage–host arms race: Shaping the evolution of microbes. Bioessays 33, 43–51. 10.1002/bies.201000071 20979102 PMC3274958

[B91] Sunagawa S. Coelho L. P. Chaffron S. Kultima J. R. Labadie K. Salazar G.. (2015). Structure and function of the global ocean microbiome. Science 348:1261359. 10.1126/science.1261359 25999513

[B92] Tatusov R. L. Galperin M. Y. Natale D. A. Koonin E. V. (2000). The COG database: a tool for genome-scale analysis of protein functions and evolution. Nucleic Acids Res. 28, 33–36. 10.1093/nar/28.1.33 10592175 PMC102395

[B93] Thingstad T. F. Bratbak G. Heldal M. (2008). Aquatic phage ecology, in Bacteriophage Ecology, ed Abedon S. T. (Cambridge: Cambridge University Press), 251–280.

[B94] Unites States Environmental Protection Agency Office of Water. (2013). Aquatic Life Ambient Water Quality Criteria for Ammonia - Freshwater, MC 4304T, 820-F-13-013. Washington, DC.

[B95] USEPA (2001). Method 1684, Total, Fixed, and Volatile Solids in Water, Solids, and Biosolids, EPA-821-R-01-015. Washington, DC.

[B96] Wang H. Holden J. Spera K. Xu X. Wang Z. Luan J.. (2013). Phosphorus fluxes at the sediment–water interface in subtropical wetlands subjected to experimental warming: a microcosm study. Chemosphere 90, 1794–1804. 10.1016/j.chemosphere.2012.08.044 22999304

[B97] Weinbauer M. G. (2004). Ecology of prokaryotic viruses. FEMS Microbiol. Rev. 28. 127–181. 10.1016/j.femsre.2003.08.001 15109783

[B98] Wichels A. Biel S. S. Gelderblom H. R. Brinkhoff T. Muyzer G. Schütt C. (1998). Bacteriophage diversity in the North Sea. Appl. Environ. Microbiol. 64, 4128–4133. 9797256 10.1128/aem.64.11.4128-4133.1998 PMC106618

[B99] Williams W. D. (2002). Environmental threats to salt lakes and the likely status of inland saline ecosystems in 2025. Environ. Conserv. 29, 154–167. 10.1017/S0376892902000103

[B100] Williamson S. J. Rusch D. B. Yooseph S. Halpern A. L. Heidelberg K. B. Glass J. I.. (2008). The Sorcerer II global Ocean sampling expedition: metagenomic characterization of viruses within aquatic microbial samples. PLoS ONE 3:e1456. 10.1371/journal.pone.0001456 18213365 PMC2186209

[B101] Winter C. Garcia J. Weinbauer M. DuBow M. Herndl G. (2014). Comparison of deep-water viromes from the Atlantic Ocean and the Mediterranean Sea. PLoS ONE 9:e100600. 10.1016/j.femsre.2003.08.001 24959907 PMC4069082

[B102] Wommack K. E. Ravel J. Hill R. T. Colwell R. R. (1999). Hybridization analysis of Chesapeake Bay virioplankton. Appl. Environ. Microbiol. 65, 241–250. 9872785 10.1128/aem.65.1.241-250.1999 PMC91008

[B103] Wurtsbaugh W. A. (1992). Food-web modification by an invertebrate predator in the Great Salt Lake (USA). Oecologia 89, 168–175. 10.1007/BF00317215 28312870

[B104] Yosef I. Manor M. Kiro R. Qimron U. (2015). Temperate and lytic bacteriophages programmed to sensitize and kill antibiotic-resistant bacteria. Proc. Natl. Acad. Sci. U.S.A. 112, 7267–7272. 10.1073/pnas.1500107112 26060300 PMC4466736

[B105] Zhou J. Bruns M. A. Tiedje J. M. (1996). DNA recovery from soils of diverse composition. Appl. Environ. Microbiol. 62, 316–322. 8593035 10.1128/aem.62.2.316-322.1996 PMC167800

[B106] Zhu W. Lomsadze A. Borodovsky M. (2010). Ab initio gene identification in metagenomic sequences. Nucleic Acids Res. 38, e132–e132. 10.1093/nar/gkq275 20403810 PMC2896542

