# Supplementary figures and images for: Insights of Phage-Host Interaction in Hypersaline Ecosystem through Metagenomics Analyses
Source: Front Microbiol. 2017 Mar 3;8:352. doi: 10.3389/fmicb.2017.00352 (PMC5334351; doi:10.3389/fmicb.2017.00352)

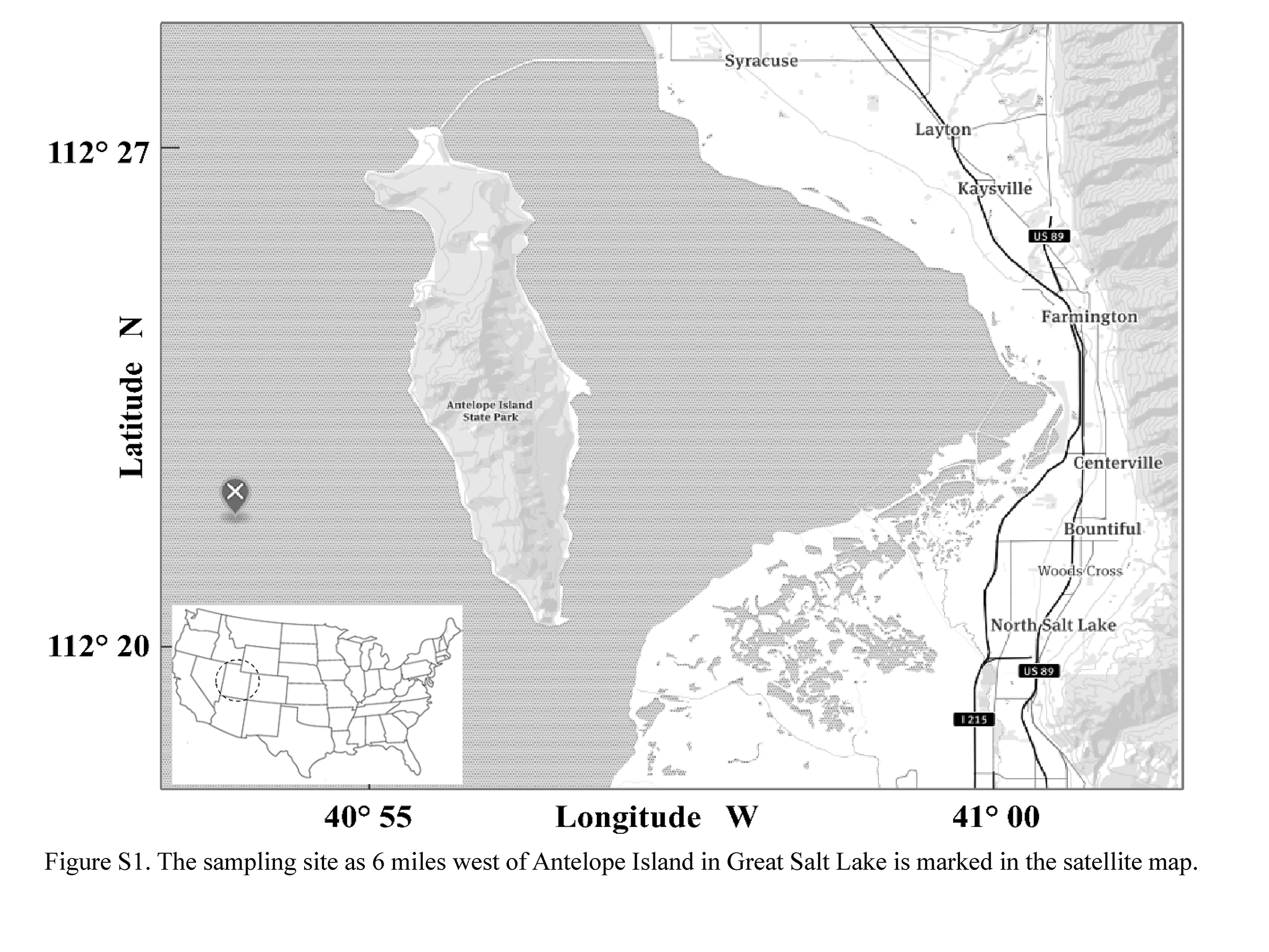

Supplement: Supplementary file 1 [file Image1.TIFF]

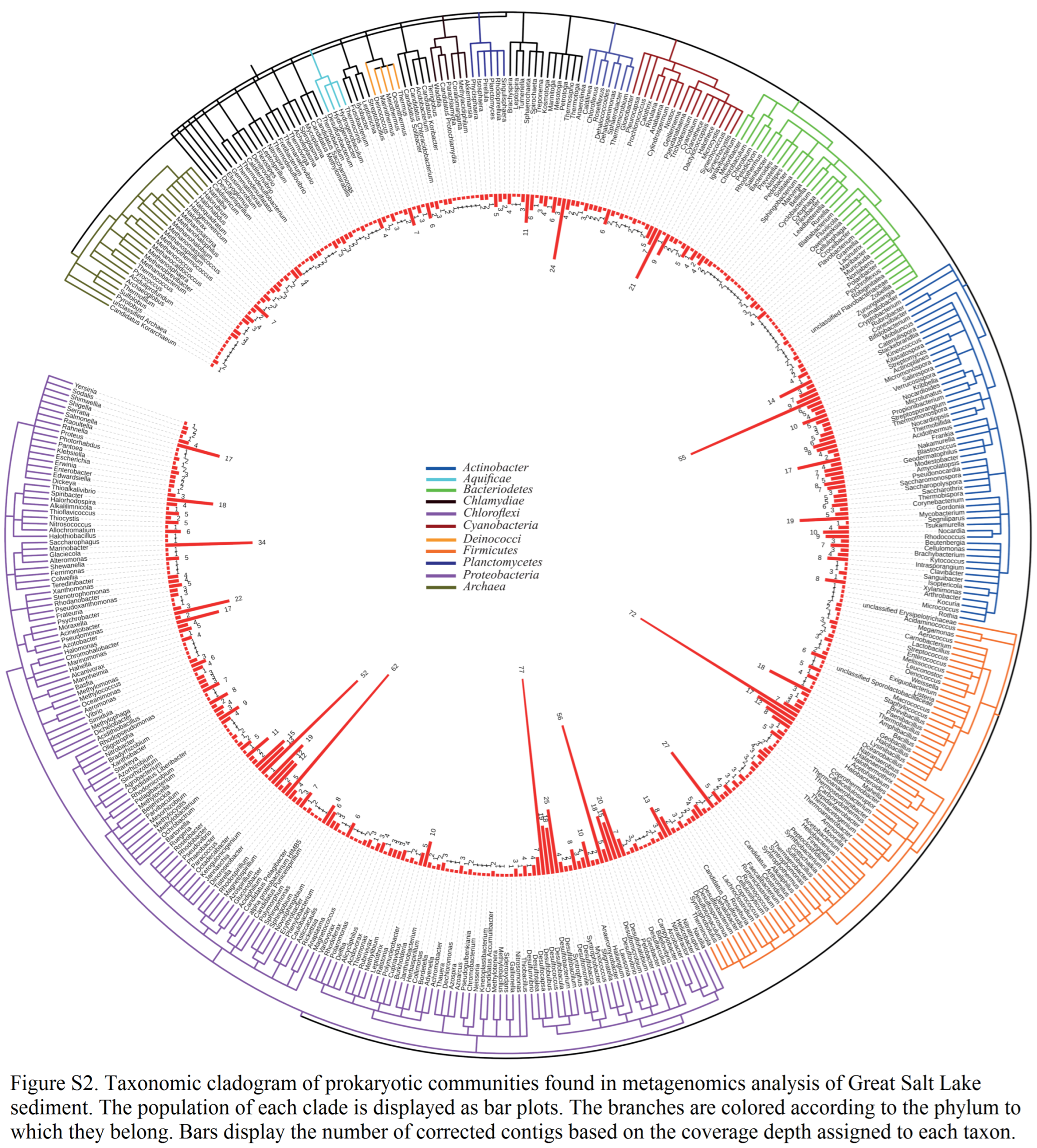

Supplement: Supplementary file 2 [file Image2.tiff]

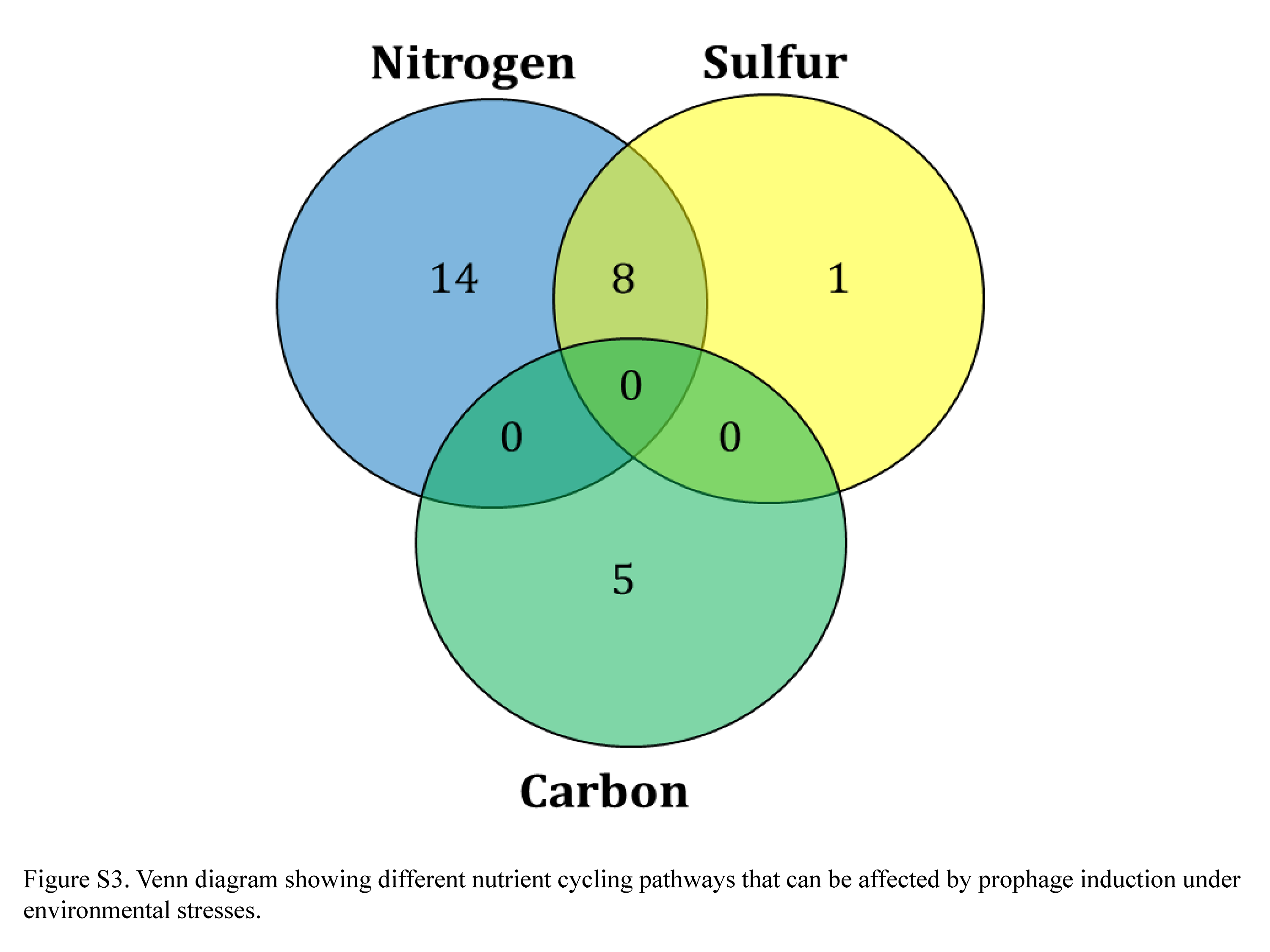

Supplement: Supplementary file 3 [file Image3.TIFF]
