# Supplementary material for: Insights of Phage-Host Interaction in Hypersaline Ecosystem through Metagenomics Analyses
Source: Front Microbiol. 2017 Mar 3;8:352. doi: 10.3389/fmicb.2017.00352 (PMC5334351; doi:10.3389/fmicb.2017.00352)
Supplement: Supplementary file 4 [file Table1.DOCX]

Table S1: Prokaryote taxonomic diversity and population found in the prokaryote contigs

| **Kingdom** | **Phylum** | | **Counts** |
| --- | --- | --- | --- |
| **Bacteria** | Actinobacteria <phylum> |  | 337 |
|  | Aquificae <phylum> |  | 8 |
|  | Bacteroidetes/Chlorobi group |  | 61 |
|  | Bacteroidetes/Chlorobi group | Bacteroidetes | 50 |
|  | Bacteroidetes/Chlorobi group | Chlorobi | 8 |
|  | Bacteroidetes/Chlorobi group | Ignavibacteriae | 3 |
|  | Caldiserica |  | 1 |
|  | Chlamydiae/Verrucomicrobia group |  | 6 |
|  | Chlamydiae/Verrucomicrobia group | Chlamydiae | 3 |
|  | Chlamydiae/Verrucomicrobia group | Verrucomicrobia | 3 |
|  | Chloroflexi |  | 40 |
|  | Chrysiogenetes <phylum> |  | 4 |
|  | Cyanobacteria |  | 67 |
|  | Deferribacteres <phylum> |  | 5 |
|  | Deinococcus-Thermus |  | 18 |
|  | Dictyoglomi |  | 4 |
|  | Elusimicrobia |  | 1 |
|  | Fibrobacteres/Acidobacteria group |  | 10 |
|  | Fibrobacteres/Acidobacteria group | Acidobacteria | 10 |
|  | Firmicutes |  | 314 |
|  | Fusobacteria |  | 7 |
|  | Gemmatimonadetes |  | 1 |
|  | Nitrospirae |  | 5 |
|  | Planctomycetes |  | 15 |
|  | Proteobacteria |  | 956 |
|  | Spirochaetes |  | 26 |
|  | Synergistetes |  | 7 |
|  | Tenericutes |  | 4 |
|  | Thermodesulfobacteria <phylum> |  | 3 |
|  | Thermotogae <phylum> |  | 18 |
|  | unclassified Bacteria |  | 4 |
|  | unclassified Bacteria | Candidate division NC10 | 2 |
|  | unclassified Bacteria | Candidatus Saccharibacteria | 1 |
|  | unclassified Bacteria | Thermobaculum | 1 |
| **Archaea** | Crenarchaeota |  | 3 |
|  | Euryarchaeota |  | 45 |
|  | Korarchaeota |  | 1 |
|  | unclassified Archaea |  | 2 |
|  | unclassified Archaea | unclassified Archaea (miscellaneous) | 2 |
| **Not assigned** | N/A |  | 119 |
