# Supplementary material for: Insights of Phage-Host Interaction in Hypersaline Ecosystem through Metagenomics Analyses
Source: Front Microbiol. 2017 Mar 3;8:352. doi: 10.3389/fmicb.2017.00352 (PMC5334351; doi:10.3389/fmicb.2017.00352)
Supplement: Supplementary file 5 [file Table2.DOCX]

Table S2: Common functional proteins in the bacteria and phage contigs

| level 1 | level 2 | level 3 | function | Abund | avg % ident | avg align len | # hits |
| --- | --- | --- | --- | --- | --- | --- | --- |
| Amino Acids and Derivatives | Aromatic amino acids and derivatives | Aromatic amino acid degradation | Anthranilate dioxygenase reductase | 1 | 92.31 | 117 | 1 |
| Carbohydrates | Central carbohydrate metabolism | Dehydrogenase complexes | Enoyl-CoA hydratase [branched-chain amino acid degradation] (EC 4.2.1.17) | 32 | 63.47 | 139.88 | 3 |
| Carbohydrates | Organic acids | Methylcitrate cycle | PrpF protein involved in 2-methylcitrate cycle | 9 | 83.95 | 111.69 | 8 |
| Carbohydrates | Organic acids | Propionate-CoA to Succinate Module | 2-methylcitrate dehydratase FeS dependent (EC 4.2.1.79) | 12 | 83.72 | 106.22 | 12 |
| Membrane Transport | - | Tricarboxylate transport system | Ammonia monooxygenase | 1 | 84.68 | 111 | 1 |
| Membrane Transport | Protein and nucleoprotein secretion system, Type IV | Vir-like type 4 secretion system | Inner membrane protein forms channel for type IV secretion of T-DNA complex, VirB8 | 10 | 94.28 | 111.43 | 4 |
| Metabolism of Aromatic Compounds | Peripheral pathways for catabolism of aromatic compounds | Benzoate degradation | Benzoate transport protein | 5 | 68.14 | 100.2 | 3 |
| Nitrogen Metabolism | - | Nitrate and nitrite ammonification | Nitrate ABC transporter, permease protein | 1 | 93.33 | 120 | 1 |
| Phages, Prophages, Transposable elements, Plasmids | Phages, Prophages | Phage replication | DNA helicase (EC 3.6.1.-), phage-associated | 52 | 69.16 | 101.21 | 6 |
| Phages, Prophages, Transposable elements, Plasmids | Plasmid related functions | Plasmid-encoded T-DNA transfer | Inner membrane protein forms channel for type IV secretion of T-DNA complex, VirB8 | 10 | 94.28 | 111.43 | 4 |
| Protein Metabolism | Protein biosynthesis | Translation elongation factors bacterial | Translation elongation factor Tu | 1131 | 82.36 | 116.75 | 188 |
| Protein Metabolism | Protein biosynthesis | tRNA aminoacylation, Thr | Threonyl-tRNA synthetase (EC 6.1.1.3), chloroplast | 41 | 62.99 | 112.17 | 1 |
| Respiration | Electron accepting reactions | Terminal cytochrome C oxidases | Cytochrome c oxidase polypeptide I (EC 1.9.3.1) | 221 | 80.66 | 101.76 | 71 |
| Respiration | Electron accepting reactions | Terminal cytochrome d ubiquinol oxidases | putative Cytochrome bd2, subunit I | 227 | 76 | 104.35 | 44 |
| Stress Response | Osmotic stress | Choline and Betaine Uptake and Betaine Biosynthesis | Glycine betaine ABC transport system, permease/glycine betaine-binding protein OpuABC | 2 | 70.75 | 106 | 1 |
| Stress Response | Oxidative stress | Rubrerythrin | Fe-S oxidoreductase-like protein in Rubrerythrin cluster | 2 | 79.67 | 100 | 2 |
