# Supplementary material for: Insights of Phage-Host Interaction in Hypersaline Ecosystem through Metagenomics Analyses
Source: Front Microbiol. 2017 Mar 3;8:352. doi: 10.3389/fmicb.2017.00352 (PMC5334351; doi:10.3389/fmicb.2017.00352)
Supplement: Supplementary file 6 [file Table3.DOCX]

Table S3: Prophage-host interaction network

| **Prophage** | **Host genera** | **Nutrient cycle involved** |
| --- | --- | --- |
| Aeromonas_phage_phiAS5 | Aeromonas | Nitrate Assimilation |
| Bacillus_phage_G | Bacillus | Nitrate Assimilation |
| Cellulophaga_phage_phiSM | Cellulomonas | Carbon Cycle |
| Cellulophaga_phage_phi10_1 | Cellulophaga | Carbon Cycle |
| Vibrio_phage_martha_12B12 | Vibrio | Sulfur Cycle |
| Vibrio_phage_martha_12B12 | Vibrio | Nitrate Assimilation |
| Vibrio_phage_pYD21_A | Vibrio | Sulfur Cycle |
| Vibrio_phage_pYD21_A | Vibrio | Nitrate Assimilation |
| Vibrio_phage_VBM1 | Vibrio | Sulfur Cycle |
| Vibrio_phage_VBM1 | Vibrio | Nitrate Assimilation |
| Vibrio_phage_ICP2 | Vibrio | Sulfur Cycle |
| Vibrio_phage_ICP2 | Vibrio | Nitrate Assimilation |
| Vibrio_phage_VvAW1 | Vibrio | Sulfur Cycle |
| Vibrio_phage_VvAW1 | Vibrio | Nitrate Assimilation |
| Burkholderia_phage_BcepC6B | Burkholderia | Nitrate Assimilation |
| Burkholderia_phage_KS9 | Burkholderia | Nitrate Assimilation |
| Burkholderia_phage_AH2 | Burkholderia | Nitrate Assimilation |
| Salmonella_phage_ST160 | Salmonella | Carbon Cycle |
| Salmonella_phage_9NA | Salmonella | Carbon Cycle |
| Escherichia_phage_TL_2011c | Escherichia | Nitrate Assimilation |
| Escherichia_phage_TL_2011c | Escherichia | Sulfur Cycle |
| Azospirillum_phage_Cd | Azospirillum | Nitrogen Fixation |
| Flavobacterium_phage_11b | Flavobacterium | Sulfur Cycle |
| Mycobacterium_phage_vB_MapS_FF47 | Mycobacterium | Sulfur Cycle |
| Mycobacterium_phage_vB_MapS_FF47 | Mycobacterium | Nitrate Assimilation |
| Rhizobium_phage_16_3 | Sinorhizobium | Nitrogen Fixation |
| Rhizobium_phage_16_3 | Sinorhizobium | Nitrate Assimilation |
| Staphylococcus_phage_SpaA1 | Staphylococcus | Carbon Cycle |
| Staphylococcus_phage_SpaA1 | Bacillus | Nitrate Assimilation |
| Thermoanaerobacterium_phage_THSA_485A | Thermoanaerobacterium | Nitrogen Fixation |
| Pseudomonas_phage_D3 | Pseudomonas | Sulfur Cycle |
| Pseudomonas_phage_D3 | Pseudomonas | Denitrification |
| Pseudomonas_phage_vB_PaeS_SCH_Ab26 | Pseudomonas | Sulfur Cycle |
| Pseudomonas_phage_vB_PaeS_SCH_Ab26 | Pseudomonas | Denitrification |
| Halovirus_HGTV_1 | Halorubrum | Nitrate Assimilation |
| Halovirus_HRTV_4 | Halorubrum | Nitrate Assimilation |
| Halovirus_HSTV_1 | Halorubrum | Nitrate Assimilation |
