# Supplementary material for: Insights of Phage-Host Interaction in Hypersaline Ecosystem through Metagenomics Analyses
Source: Front Microbiol. 2017 Mar 3;8:352. doi: 10.3389/fmicb.2017.00352 (PMC5334351; doi:10.3389/fmicb.2017.00352)
Supplement: Supplementary file 7 [file Table4.DOCX]

Table S4: The 40 most abundant bacterial taxonomic diversity and relative abundance in genus level

| **Bacterial taxon (genus level)** | **Taxon abundance** |
| --- | --- |
| Clostridium | 56290 |
| Anaerolinea | 39327 |
| Bacillus | 38115 |
| Bacteroides | 35600 |
| Desulfovibrio | 35582 |
| Halothermothrix | 32265 |
| unclassified (derived from unclassified sequences) | 28114 |
| Burkholderia | 26599 |
| Desulfococcus | 24254 |
| Halanaerobium | 24089 |
| Marinobacter | 22445 |
| Pseudomonas | 22162 |
| Geobacter | 21117 |
| Desulfatibacillum | 19901 |
| Desulfobacterium | 19673 |
| Roseobacter | 19294 |
| Roseovarius | 17210 |
| Ruegeria | 16949 |
| Desulfohalobium | 16485 |
| Streptomyces | 16164 |
| unclassified (derived from Deltaproteobacteria) | 16008 |
| Roseiflexus | 14703 |
| Lactobacillus | 14444 |
| Synechococcus | 14123 |
| Desulfotomaculum | 13841 |
| Kineococcus | 13832 |
| Escherichia | 13684 |
| Syntrophobacter | 13611 |
| Streptococcus | 13073 |
| Spirochaeta | 12615 |
| Rhodobacter | 12387 |
| Syntrophus | 12267 |
| Salmonella | 11707 |
| Methylobacterium | 11294 |
| Shewanella | 10849 |
| Halarsenatibacter | 10793 |
| Vibrio | 10225 |
| Paenibacillus | 9743 |
| Pelobacter | 9019 |
